# Rabies control in Bangladesh and prediction of human rabies cases by 2030: a One Health approach

**DOI:** 10.1016/j.lansea.2024.100452

**Published:** 2024-07-23

**Authors:** Sumon Ghosh, Mohammad Nayeem Hasan, Nirmalendu Deb Nath, Najmul Haider, Daleniece Higgins Jones, Md. Kamrul Islam, M. Mujibur Rahaman, Hasan Sayedul Mursalin, Nadim Mahmud, Md. Kamruzzaman, Md. Fazlay Rabby, Shotabdi Kar, Sayed Mohammed Ullah, Md. Rashed Ali Shah, Afsana Akter Jahan, Md. Sohel Rana, Sukanta Chowdhury, Md. Jamal Uddin, Thankam S. Sunil, Be-Nazir Ahmed, Umme Ruman Siddiqui, S.M. Golam Kaisar, Md. Nazmul Islam

**Affiliations:** a Department of Public Health, College of Education, Health, and Human Sciences, The University of Tennessee, Knoxville, TN 37996, USA; b Disease Control Unit, Communicable Disease Control, Directorate General of Health Services, Ministry of Health and Family Welfare, Bangladesh; c Department of Statistics, Shahjalal University of Science and Technology, Sylhet, Bangladesh; d Department of Biomedical and Diagnostic Sciences, The University of Tennessee, Knoxville, USA; e School of Life Sciences, Keele University, Staffordshire, ST5 5BG, United Kingdom; f Livestock Research Institute, Department of Livestock Services, Ministry of Fisheries and Livestock, Dhaka, Bangladesh; g International Centre for Diarrheal Disease Research, Bangladesh (icddr,b), Dhaka, Bangladesh; h Faculty of Graduate Studies, Daffodil International University, Dhaka, 1216, Bangladesh

**Keywords:** Human rabies, Time-series forecasting models, Mass dog vaccination, Bangladesh

## Abstract

**Background:**

Bangladesh is making progress toward achieving zero dog-mediated rabies deaths by 2030, a global goal set in 2015.

**Methods:**

Drawing from multiple datasets, including patient immunisation record books and mass dog vaccination (MDV) databases, we conducted a comprehensive analysis between 2011 and 2023 to understand the effectiveness of rabies control programmes and predict human rabies cases in Bangladesh by 2030 using time-series forecasting models. We also compared rabies virus sequences from GenBank in Bangladesh and other South Asian countries.

**Findings:**

The estimated dog population in Bangladesh was determined to be 1,668,140, with an average dog population density of 12.83 dogs/km^2^ (95% CI 11.14–14.53) and a human-to-dog ratio of 86.70 (95% CI 76.60–96.80). The MDV campaign has led to the vaccination of an average of 21,295 dogs (95% CI 18,654–23,935) per district annually out of an estimated 26,065 dogs (95% CI 22,898–29,230). A declining trend in predicted and observed human rabies cases has been identified, suggesting that Bangladesh is poised to make substantial progress towards achieving the ‘Zero by 30’ goal, provided the current trajectory continues. The phylogenetic analysis shows that rabies viruses in Bangladesh belong to the Arctic-like-1 group, which differs from those in Bhutan despite sharing a common ancestor.

**Interpretation:**

Bangladesh's One Health approach demonstrated that an increase in MDV and anti-rabies vaccine (ARV) resulted in a decline in the relative risk of human rabies cases, indicating that eliminating dog-mediated human rabies could be achievable.

**Funding:**

The study was supported by the Communicable Disease Control (CDC) Division of the Directorate General of Health Services (DGHS) of the People's Republic of Bangladesh.

**Research in context**

**Evidence before this study**

In 2011, Bangladesh launched the National Rabies Elimination Programme to eliminate dog-mediated human rabies through a one-health approach and is making strides towards achieving the global strategic plan set in 2015 to reach the goal of zero rabies deaths by 2030. The role of mass dog vaccination (MDV) in reducing dog-mediated human rabies deaths in Bangladesh has been published before. However, the study did not report any prediction of human rabies cases in Bangladesh aligned with the ‘Zero by 30’ goal using time-series forecasting models. We searched PubMed on July 31, 2023, with the terms “Human Rabies,” “Time-series Forecasting Models,” “Mass Dog Vaccination,” and “Bangladesh” with no date or language restrictions, and found no previous publications.

**Added value of this study**

This study is the first in Bangladesh to use time-series forecasting models to estimate the future incidence of human deaths from dog-mediated rabies. Bangladesh has made significant progress in reducing human deaths from dog-mediated rabies through a One Health approach; a decreasing trend in predicted and observed cases of human rabies has been identified. If the current trend continues, it is projected that Bangladesh will have zero human rabies cases by 2030.

**Implications of all the available evidence**

In 2015, the global community issued a call to action with the goal of achieving zero human deaths from dog-mediated rabies by 2030. The Global Alliance for Rabies Control (GARC) and the Tripartite, consisting of the Food and Agriculture Organization of the United Nations (FAO), the World Organization for Animal Health (WOAH), and the WHO, convened in 2018 to establish the United Against Rabies Forum. This collaboration led to introducing the ‘Zero by 30’ initiative, which outlines a Global Strategic Plan to end human deaths from dog-mediated rabies by 2030.

The initiative embodies a model for One Health collaboration and aligns with the UN Sustainable Development Goals. The effectiveness of the Mass Dog Vaccination (MDV) strategy in interrupting dog-to-dog or dog-to-human transmission of the rabies virus is well-documented. Achieving a 70% coverage rate in annual pulse vaccination campaigns is expected to result in a substantial reduction in the incidence of rabies cases among dogs. Sustaining this high level of coverage over consecutive years holds promise for the regional elimination of rabies. Regular evaluation of regional and global data is vital to monitor progress toward the goal of Zero by 30. Rabies virus transmission models can aid countries in their efforts to eliminate human deaths from dog-mediated rabies by 2030. While Bangladesh is marching towards the shared goal of Zero by 30, information on rabies trends and forecasting is crucial.

The findings of this study can aid the development of policy decisions to support national rabies control plans in Bangladesh and other similarly socioeconomically situated countries, accelerating the progress towards eliminating dog-mediated human rabies globally and achieving the zero by 30 target.

## Introduction

Rabies, a zoonotic viral disease primarily transmitted to humans through the bite of infected dogs, kills an estimated 59,000 people each year worldwide and has the highest case fatality rate of any infectious disease.^1^^,^^2^ Annually, over 29 million individuals worldwide receive post-exposure prophylaxis (PEP), mainly in Asia and Sub-Saharan Africa, resulting in a substantial economic loss of US$ 8.6 billion alongside unquantified psychological trauma for affected individuals and communities.^1^ The WHO, the World Organisation for Animal Health (WOAH), the Food and Agriculture Organization of the United Nations (FAO), and the Global Alliance for Rabies Control (GARC)–collectively known as “United Against Rabies”–have globally announced a call to eliminate dog-mediated human rabies by 2030 (“Zero by 30”) and are committed to providing technical support to achieve this goal.^3^

Rabies has high public health consequences for Bangladesh. It ranks third among rabies-endemic countries in the world in terms of human rabies deaths, about 96% of which are attributable to dogs.^4^^,^^5^ The Government of Bangladesh (GOB) launched the National Rabies Elimination Programme in 2011 and is taking necessary actions to achieve the ‘Zero by 30’ goal through practical progress in rabies control.^6^ GOB was instrumental in driving the programme forward by implementing strategies, including advocacy, communication, and social mobilization (ACSM), modern treatment for dog bites, mass dog vaccination (MDV), and dog population management.^7^ This programme reduced the number of deaths from human rabies by approximately 50% in recent years in Bangladesh.^8^

The early administration of human PEP can prevent rabies-related deaths, but treating people (a dead-end host) after being bitten has little effect on the incidence of rabies in the canine reservoir population, leaving additional members of the community at risk of contracting the infection.^9^ For over a century, it has been known that MDV may successfully eliminate rabies from the reservoir canine population, preventing virus transmission to humans and eliminating dog-mediated rabies in many regions.^9^^,^^10^ In a One Health approach, modern rabies management emphasises the necessity of attaining zoonotic disease prevention and control by considering human, animal, and environmental factors.^11^ MDV is an effective method to stop dog-to-dog or dog-to-human rabies virus transmission. If annual pulse vaccination campaigns achieve over 70% coverage, rabies incidence in dogs is likely to be significantly reduced, and regional elimination is possible if this coverage is sustained over several years.^12^^,^^13^

It is crucial to evaluate the available data regularly at regional and global levels to adequately track the advancement toward the eventual goal of ‘Zero by 30’. Accurately predicting infectious disease trends is essential for optimal resource allocation and future control measures.^14^ Historical data are typically used to forecast future events in epidemiology, especially when the size of the susceptible population is not defined.^15^ Time series analysis is a crucial tool for forecasting infectious disease with high accuracy, which helps develop effective disease control strategies and assess the impact of interventions.^16^ Given the global goal of eliminating human deaths from dog-mediated rabies by 2030, models of rabies virus transmission have the potential to inform control efforts as countries continue to work toward elimination. While Bangladesh is marching towards the shared goal of ‘Zero by 30’, information on rabies trends and forecasting is crucial. We aimed to examine trends and forecast human rabies cases concerning the usage of MDV and the human anti-rabies vaccine (ARV) in Bangladesh. Furthermore, we have compared the phylogenetic analyses of rabies virus sequences reported in Bangladesh and other south Asian countries extracted from GenBank.

## Methods

### Data source

We obtained data from patient immunisation record books maintained at the National Rabies Prevention and Control Centre (NRPCC) located in the Infectious Disease Hospital (IDH) in Dhaka, Bangladesh, from 2011 to 2023. The NRPCC, Bangladesh's primary referral center for animal bites and rabies, offers free vaccination and treatment for most cases nationwide, with the Bangladesh Institute of Tropical and Infectious Diseases (BITID) in Chittagong serving as the secondary referral center for rabies after NRPCC.^6^ Besides NRPCC and BITID, 66 public District Rabies Prevention and Control Centres (DRPCCs) exist, with at least one centre in each of the 64 districts, which provides free PEP and treatment to individuals who have suffered animal bites. The data included demographic information of patients, details on the type and dosages of vaccines and rabies immunoglobulin (RIG) administered to bite victims and reported cases of rabies in humans. For data regarding MDV, we used the MDV database of the Communicable Disease Control Division (CDC), Bangladesh's Directorate General of Health Services (DGHS), from November 2011 to April 2023. The database includes data on all estimated and vaccinated dogs, human-to-dog ratios, and vaccination coverage in all 64 districts of Bangladesh.

### Human rabies surveillance

Human rabies surveillance in Bangladesh was conducted by CDC DGHS, with local health officials reporting epidemiological information on animal exposures and rabies cases to central authorities. Data was primarily collected on paper, with limited disaggregation and manual transfer into the national District Health Information (DHIS2) system. Laboratory confirmation of human rabies cases needed to be improved due to cultural sensitivity and logistical constraints. Diagnosis was mainly based on clinical symptoms and verbal autopsy, highlighting the need for improved surveillance accuracy. The DGHS has a central core committee overseeing human rabies surveillance activities. CDC DGHS led this committee, and additional personnel were included, such as the deputy director, deputy programme manager, and surveillance medical officer. The Upazila (sub-district) Rapid Response Team (URRT), comprised of medical professionals, health assistants, and other health personnel, has been investigating reports of patients who have had contact with animals (typically bites or scratches) with information shared with central rabies teams. The CDC and NRPCC's central rabies teams and the intermediate-level (Civil Surgeon) rabies teams have regularly received information and aggregate statistics from the local level.

### MDV

The CDC DGHS coordinated the MDV programmes as part of the national rabies elimination programmes. They also supplied required dog rabies vaccines for MDV. Along with the DGHS, the MDV programmes in Bangladesh also received contributions in various forms from the Department of Livestock Services (DLS), the Local Government Division, the Education sectors, and non-governmental organisations (NGOs) or development organisations.^6^ The MDV activities were conducted methodically, starting with establishing the programme date by contacting the responsible officials from the CDC DGHS for the specific area (district or sub-district), and 7–9 days were allocated for the campaign in each area. This involved one day for advocacy and microplanning, another day for animal control staff (ACS) training, and a 4–6-day vaccination campaign. Necessary correspondence was sent to confirm the program's workforce and to gather and dispatch the required logistics to the designated area. Following this, an Advocacy Meeting was convened to inform key individuals, resource persons, and journalists in the specified area about the programme. Microplanning was then undertaken, allocating work areas by date and developing detailed plans for each location within the designated area of the MDV programme. Subsequently, Animal Control Staff Training was conducted, encompassing a one-day session to train the workforce on the MDV programme, featuring hands-on training for dog catchers, surveyors, and vaccinators. The training was led by District Livestock Officers or/Upazila (Sub-district) Livestock Officers and facilitated by authorised personnel from CDC DGHS. Overall coordination was overseen by the Civil Surgeon (District)/Upazila Health and Family Planning Officer. The vaccination programme, implemented over 5–6 days, applied the One Health concept, bringing together expertise from various sectors. A typical MDV team comprised six members from different sectors: two expert dog catchers, one local dog catcher, one surveyor/data collector from the health sector, one vaccinator from the DLS, and one porter from the local government.^17^ The vaccination was conducted employing the Catch-vaccinate-release (CVR) method. Each dog was administered a 1 ml dose of the rabies vaccination (CaniShot RV-K -(CAVAC); CANVAC® R–Dyntec; Rabisin®–Merial), delivered either subcutaneously or intramuscularly, based on the animal's posture and mode of restraint. A water-soluble and non-toxic rhodamine dye was used to mark each dog to make it easier to identify the vaccination status on post-vaccination surveys. Required data were recorded in the KoBo Toolbox mobile app as well as in the paper sheet.^18^

After the campaign, a post-vaccination survey (PVS) was carried out using the sight-resight method to determine vaccination coverage and estimate the dog population.

*Dog Population Estimation:* ^19^ ^,^ ^20^

We used the following formula to estimate the vaccination coverage:

$$\frac{\text{Number}\;\text{of}\;\text{marked}\;\text{dogs}\;\text{seen}\;\times \;100}{\text{Total}\;\text{number}\;\text{of}\;\text{dogs}\;\text{seen}}$$

The following formula was used to estimate the dog population size:

$$\frac{\text{Number}\;\text{of}\;\text{dogs}\;\text{vaccinated}\;\text{and}\;\text{marked}\;\times \;\text{Total}\;\text{number}\;\text{of}\;\text{dogs}\;\text{seen}}{\text{Number}\;\text{of}\;\text{marked}\;\text{dogs}\;\text{seen}}$$

In Bangladesh, MDV campaigns were usually carried out annually in specific areas, assuming immunity against rabies following vaccination would endure for a minimum of one year.^6^^,^^21^ The initiative was rolled out nationwide in various phases to conduct at least three rounds of MDV nationwide. The potential for dogs to receive multiple vaccinations was considered. Although we recognise the potential for dogs to relocate to new areas or sub-districts, this slight possibility was disregarded due to insufficient data availability for consideration of such scenarios.

To determine the district-wise average vaccination coverage of dogs in Bangladesh, we divided the number of vaccinated dogs in each district by the estimated dog population and then multiplied the result by 100. To address potential errors related to double counting, we considered the average estimated and vaccinated dog populations, as well as the number of rounds of MDV in each district. The frequency of vaccination rounds (MDV) varied across districts in Bangladesh, as outlined in the government's strategic plan, with some districts receiving one round, others receiving two, and some receiving three rounds. Additionally, we calculated the district-wise human-to-dog ratio by dividing the human population by the estimated dog population in each district. Furthermore, we calculated the district-wise dog population density by dividing the estimated dog population by the area of each respective district in Bangladesh (Supplementary file, page 6–9).

### Statistical analysis

Hierarchical clustering using Pearson correlation coefficients was used to visualise associations between variables (MDV, ARV, and the year the vaccinations occurred) and possible clusters using pairwise comparisons to adjust correlations to measure dissimilarity and the distribution function in R software.

#### Time series analysis

Earlier observations potentially influence any variable measured across time, an essential aspect of time-series analysis (autocorrelation). Time-series models use previous observations to forecast future behavior to take advantage of these linkages. This is the significant difference between time-series analysis and conventional statistical tests for assessing change, such as regression analysis, which relies on independent variable variation to explain outcome changes.^22^ A time-series forecasting model called the seasonal autoregressive integrated moving average (SARIMA) model was employed to forecast rabies cases based on monthly data.^23^ This model combines various components, namely the trend cycle, seasonality, and error, and can be represented by the following equation:

$$Z_{t}=T_{t}\;+\;S_{t}\;+\;E_{t}$$

Here, Z_t_ refers to the observed data, T_t_ represents the trend-cycle component, S_t_ represents the seasonal component, and E_t_ accounts for the portion of data not accounted for by the model in a given period, also known as the error or random residual.

As the primary outcome variable for rabies cases is influenced by past reported cases with some seasonality, we chose the SARIMA model (time-series events). The SARIMA model is a data-focused, exploratory technique that enables the user to construct an appropriate model based on the data's actual structure.^23^^,^^24^ This model attempts to extract regional trends while filtering out high-frequency noise from the data and assumes a linear correlation between the time series values.^25^ By updating the model to forecast the system's future state based on current occurrences, SARIMA models have the advantage of adapting to dynamically oriented systems that change over time. The R package “forecast” was used to run the SARIMA model for this study.^26^ We predicted trends for the upcoming seven years (up to 2030) using SARIMA time series models with the data on rabies cases and showed them in the figure.

The Box and Jenkins approach was utilised in formulating the seasonal autoregressive integrated moving average (SARIMA) model, which is intended for analysing stationary time series data.^23^^,^^27^^,^^28^ The SARIMA model is characterised by the notation SARIMA (p,d,q) (P, D, Q)_m_, where p and P represent the order of the non-seasonal autoregressive polynomial and seasonal autoregressive polynomial, respectively. Similarly, q and Q denote the order of the non-seasonal moving average polynomial and seasonal moving average polynomial, respectively. The autoregressive and moving average orders were selected by examining the autocorrelation function (ACF) and partial autocorrelation function (PACF). Furthermore, d and D represent the orders of differentiation applied to the time series to achieve stationarity. A SARIMA (p,d,q) (P, D, Q)_m_ process refers to an autoregressive moving average (SARMA) model that has been differenced d and D times to obtain stationarity for monthly data.

Numerous researchers have recently used time series to study the trend of rabies (including additive models,^29^ auto-regressive time series models,^30^ and wavelet time series models^31^); however, most of them use the ARIMA model with one-time series because it is a helpful method for analysing time series with one-time series in systems.^32^^,^^33^ Hence we used the SARIMA model to capture the seasonality of the outcome variable. SARIMA models accept a direct relationship between the time-series values and seek to leverage these straight circumstances in perceptions to extract nearby designs while reducing high-frequency turbulence. However, as SARIMA only considers one variable, it could not provide light on the connections between system variables. As part of our analysis, we must acknowledge a limitation we encountered: the presence of negative values within the confidence intervals of forecasted values. Therefore, we addressed this limitation by replacing negative values within the confidence intervals with zero while leaving the forecasted values unchanged.

We employed count time series following generalized linear models (TSGLM) to examine the relationship between our study's outcome variable, which consists of count data, and other variables. This class of models offers excellent flexibility and can effectively capture serial correlation while maintaining simplicity. The past observations, potential effects of covariates, and previous values are linked to the conditional mean of the observed process. Our research mainly concentrated on models that used the logarithmic link function as the conditional distribution conformed to a Poisson regression model.

In this study, MDV and ARV were considered explanatory variables in the TSGLM model. The study utilised the incidence risk ratio (IRR) to present results, with 95% confidence intervals established at the <0.05 (5%) significance level. However, a somewhat arbitrary p-value threshold of <0.10 (10%) was also considered for interpretation purposes. It is important to note that while a p-value may be extremely small, it does not definitively confirm the alternative hypothesis or the null hypothesis.^34^ Additionally, the p-value alone does not convey the magnitude of the difference between groups. In cases where the sample size is large or measurements are highly precise, statistical significance may be achieved even if the difference lacks clinical relevance.^35^ The ‘tsglm’ packages from R software were used for TSGLM analysis.

#### Empirical evaluation

The relevance of the predictions empirically assesses the SARIMA model; we analysed and compared the performance of this time series model using some of the generally used measures, including the Akaike information criterion (AIC), the Bayesian information criterion (BIC), the Akaike information criterion with correction (AICc), coefficient of determination (R^2^), root mean square error (RMSE), and mean absolute error (MAE). Models associated with smaller values of a given AIC, BIC, AICc, RMSE, and MAE are ranked higher than those associated with larger values, and the larger the value of R^2^ indicates better fit, the absolute value of the criterion being irrelevant.^36^^,^^37^ Additionally, AIC, BIC, RMSE, and R^2^ values were assessed to evaluate the performance and predictive capacity of the TSGLM model.

### Cartographic display

The cartographic boundary files, which are used to create maps, were downloaded from the DIVA-GIS.^38^ All cartographic displays were performed in ArcGIS version 10.8.1.^39^ Choropleth maps were used to display the district-level geographic distribution of rabies cases, human-to-dog ratios, number of vaccinated dogs, and percentage of vaccination coverage using Jenk's optimization classification scheme.

### Phylogenetic analysis

We used the complete genome sequences of the N gene of the arctic-like rabies virus from Bangladesh (GenBank accession number AB699214.1) and blasted in the National Centre for Biotechnology Information (NCBI) to generate a phylogenetic tree. Then, we downloaded 68 complete genome sequences of the N gene from Bangladesh, India, Pakistan, Nepal, Afghanistan, and Bhutan. The sequences were aligned by the MUSCLE method in MEGA 11 software. The N sequences' phylogenetic relationship was determined using the neighbor-joining method in MEGA 11. The maximum likelihood phylogenetic trees of the N segment were generated using the Hasegawa-Kishino-YanoJukes model with a bootstrap value of 1000.^40^^,^^41^ Finally, the phylogenetic tree was visualised in FigTree version v1.4.3. The viruses from Bangladesh were labeled red.

### Role of the funding source

The study was supported by the Communicable Disease Control (CDC) Division of the Directorate General of Health Services (DGHS) of Bangladesh, which was involved in the study design but played no role in its analysis, writeup, or publication.

## Results

### MDV, ARV, and human rabies

**Fig. 1:**
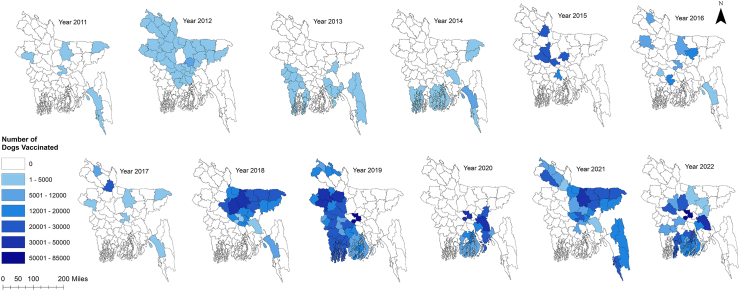
Year-wise scaling up mass dog vaccination (MDV) in different districts of Bangladesh, 2011–2022. Map of Bangladesh showing the number of dogs vaccinated in different districts of Bangladesh starting from 2011.

**Fig. 2:**
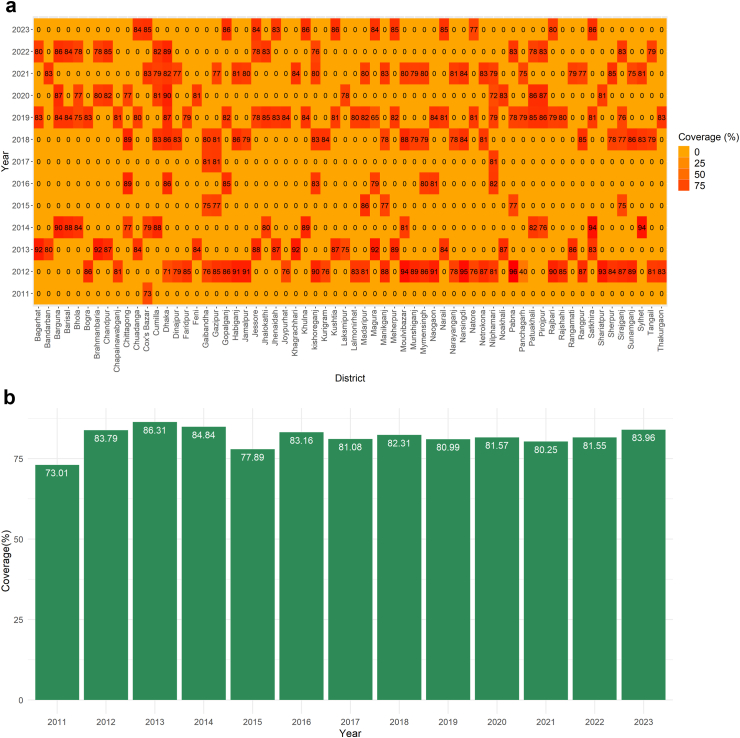

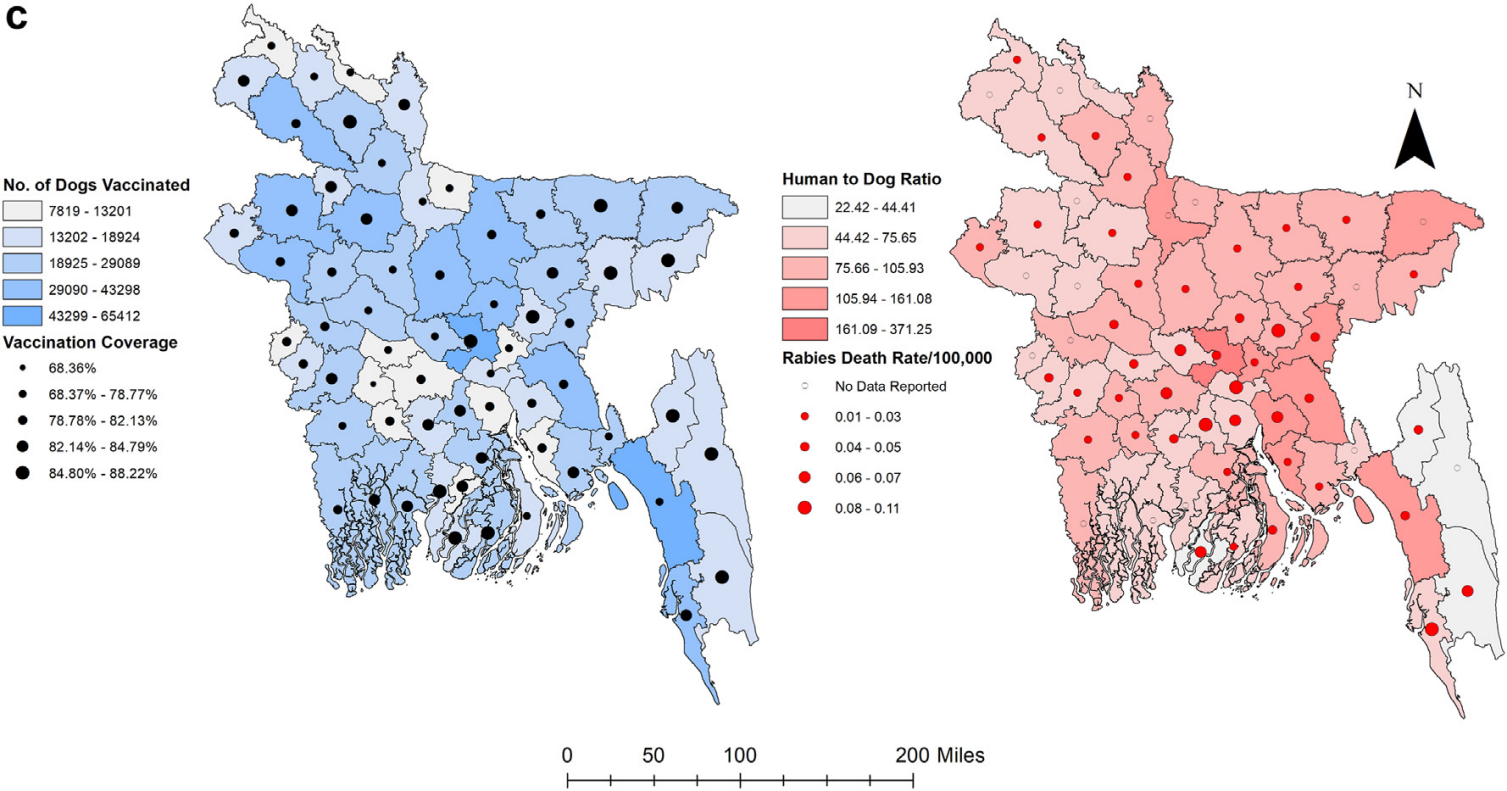
(a): Heat map of year-wise vaccination coverage (%) in different districts of Bangladesh, (b): Year-wise vaccination coverage (%) in different districts of Bangladesh, (c): Map of Bangladesh shows dog vaccination and estimated human-to-dog ratio by district. Left: Showing the number of dogs vaccinated (blue shadow) with vaccination coverage (black circles). Right: Showing Human to dog ratio (red shadow) with human rabies death rate per 100,000 population (red circle).

We subjected 12 years of MDV data maintained at Bangladesh's Directorate General of Health Services (DGHS). Bangladesh's MDV programme began in 2011 in the municipality of Cox's Bazar on the country's southeast coast. During this period, MDV was conducted at least once per year and scaled up for at least one round in all 64 country districts. Forty-five districts have already completed their second round of vaccination, while eight districts have completed their third round. From 2011 onward, an upward trend of MDV was found, while the highest number of dogs was vaccinated in 2019 (n = 625,208) (Fig. 1, Supplementary Figure S1). Under the MDV campaign, an average of 21,295 dogs (95% CI 18,654–23,935) were vaccinated per district annually, out of an estimated 26,065 dogs (95% CI 22,898–29,230), where vaccination coverage exceeded the threshold level in almost all districts [Fig. 2a, b]. The estimated dog population in Bangladesh was determined to be 1,668,140, with an average dog population density of 12.83 dogs/km^2^ (95% CI 11.14, 14.53) and a human-to-dog ratio of 86.70 (95% CI 76.60, 96.80) [Fig. 2c, Supplementary Figure S2]. From 2011 to 2023, a total of 2,730,829 doses of ARV were used to manage the animal bite cases at the National Rabies Prevention and Control Centre (NRPCC), the Bangladesh Institute of Tropical and Infectious Diseases (BITID), and the District Rabies Prevention and Control Centres (DRPCCs) of Bangladesh with an average of 18451.55 doses ARV used each month [Supplementary Figure S3a, b]. In total, 879 cases of human rabies were reported in Bangladesh between 2011 and 2023, with an average of 5.63 cases recorded monthly (0.0034 cases per 100,000 population) and average sixty-six cases recorded annually (0.04 cases per 100,000 population). The highest number of cases was reported in 2014 (106 cases, 6.8 cases per 10 million people). The districts in the middle of the country (the capital and nearby districts) saw the highest number of cases [Supplementary Figure S4a]. We also found an increasing trend of ARV utilisation and dog vaccination as well as a decreasing trend of human rabies cases in Bangladesh from 2011 to 2022 [Supplementary Figure S4b].

**Fig. 3:**
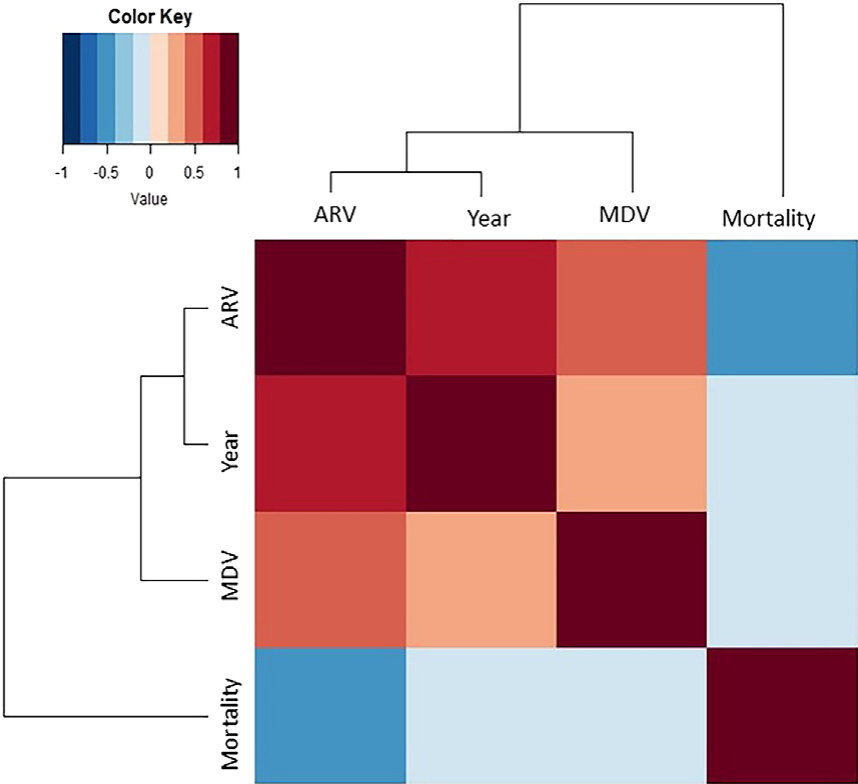
Correlation matrix heatmap with dendrogram depicting associations between variables. The length of the dendrogram branches represents the distance between variables from the Pearson correlation. Correlations range from 1 for perfectly correlated variables to −1 for negatively correlated variables. Mortality: human deaths, MDV: dog rabies vaccinations, year: year of vaccination, and ARV: human rabies vaccinations).

Two main clusters were revealed by the dendrogram of the rabies virus in Bangladesh (Fig. 3). The left main branch displays the first strong cluster. This cluster exposed strong to moderate positive correlations between three variables (MDV, ARV, and the year the vaccinations occurred). We have found strong positive correlations between years of MDV and ARV (correlation coefficient: 0.60). Weak positive correlations have been found between ARV and MDV (correlation coefficient: 0.32), whereas MDV and year of vaccination showed moderate positive correlations (correlation coefficient: 0.41). The following central cluster is the right main branch, with moderate negative correlations for human rabies death. Human rabies death and year of vaccination have a moderate negative correlation (Correlation coefficient: −0.56), whereas MDV and ARV and human rabies death were comparatively found to have a weaker negative correlation (correlation coefficient: −0.20; −0.19), respectively.

### Phylogenetic tree

**Fig. 4:**
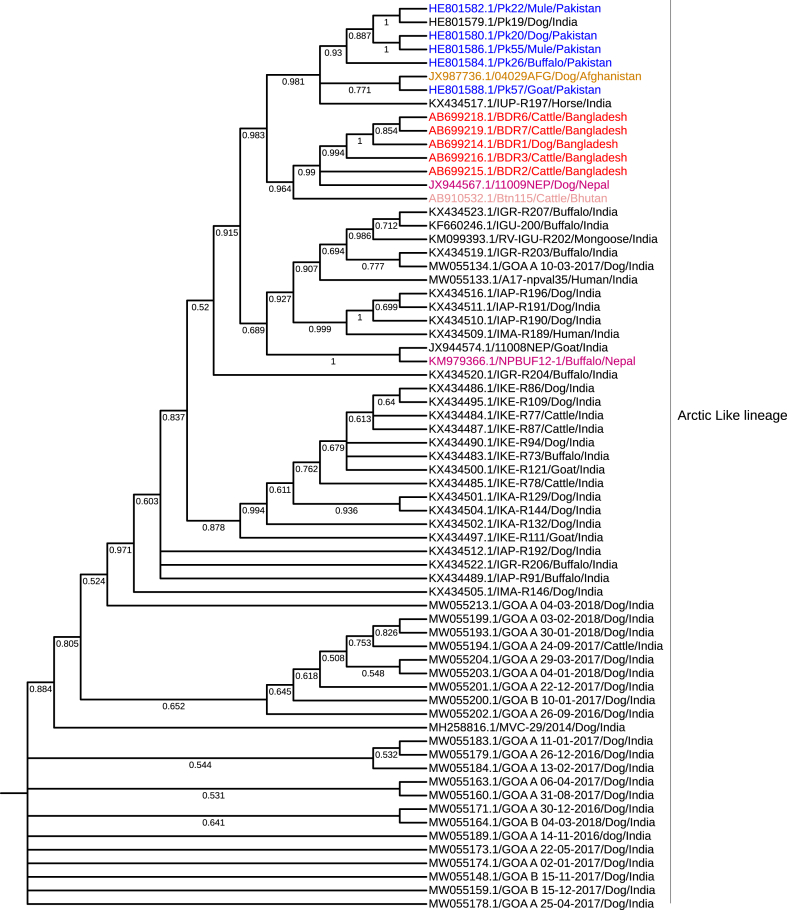
Phylogeographic distribution of canine rabies cases. Phylogenetic analysis of 68 complete genome sequences of the N gene from Bangladesh, India, Pakistan, Nepal, Afghanistan, and Bhutan was used. Complete genome sequences of the N gene of arctic-like rabies virus from Bangladesh (GenBank accession number AB699214.1) and blasted in the National Centre for Biotechnology Information (NCBI) generated a phylogenetic tree. The maximum likelihood phylogenetic trees of the N segment were generated using the Hasegawa-Kishino-YanoJukes model with a bootstrap value of 1000. The viruses from Bangladesh were labeled red.

Coordinated rabies control efforts in the area continue to be hampered by a need for more knowledge regarding the phylogenetic relationships between the rabies virus in Bangladesh and viruses in other countries. To explain its epidemiologic relationships, origin, and transmission dynamics, a phylogenetic analysis was performed to characterise the rabies virus circulating in Bangladesh and determine its relationship with viruses in neighboring countries. The phylogenetic tree revealed that the rabies viruses in Bangladesh are members of the Arctic/Arctic-like virus, also called Arctic-like-1. N gene sequences from rabies viruses in Bhutan and Bangladesh show a strong link, suggesting that they shared an ancestor and developed into distinct strains. The exact lineage circulated in other countries in this region, including India, Nepal, Pakistan, and Afghanistan, as shown by the tree samples clustered separately from sequences generated in other studies (Fig. 4).

### Time-series analysis

**Fig. 5:**
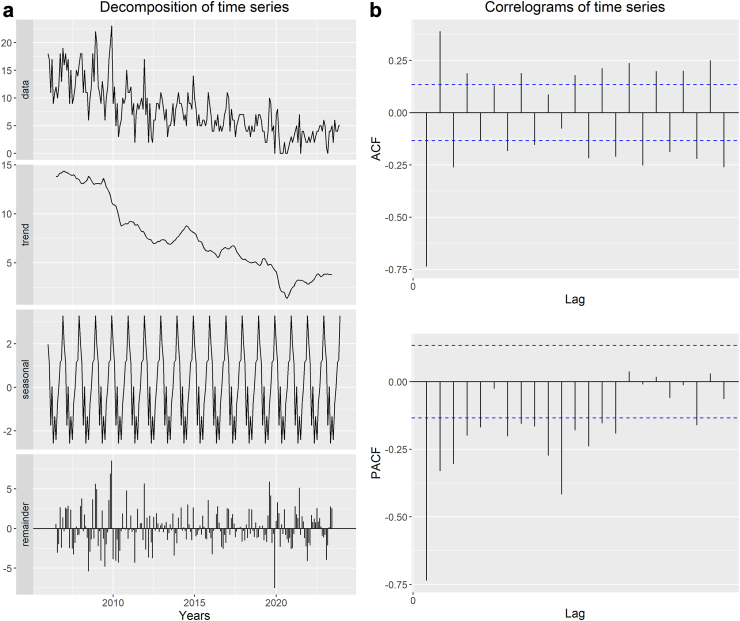

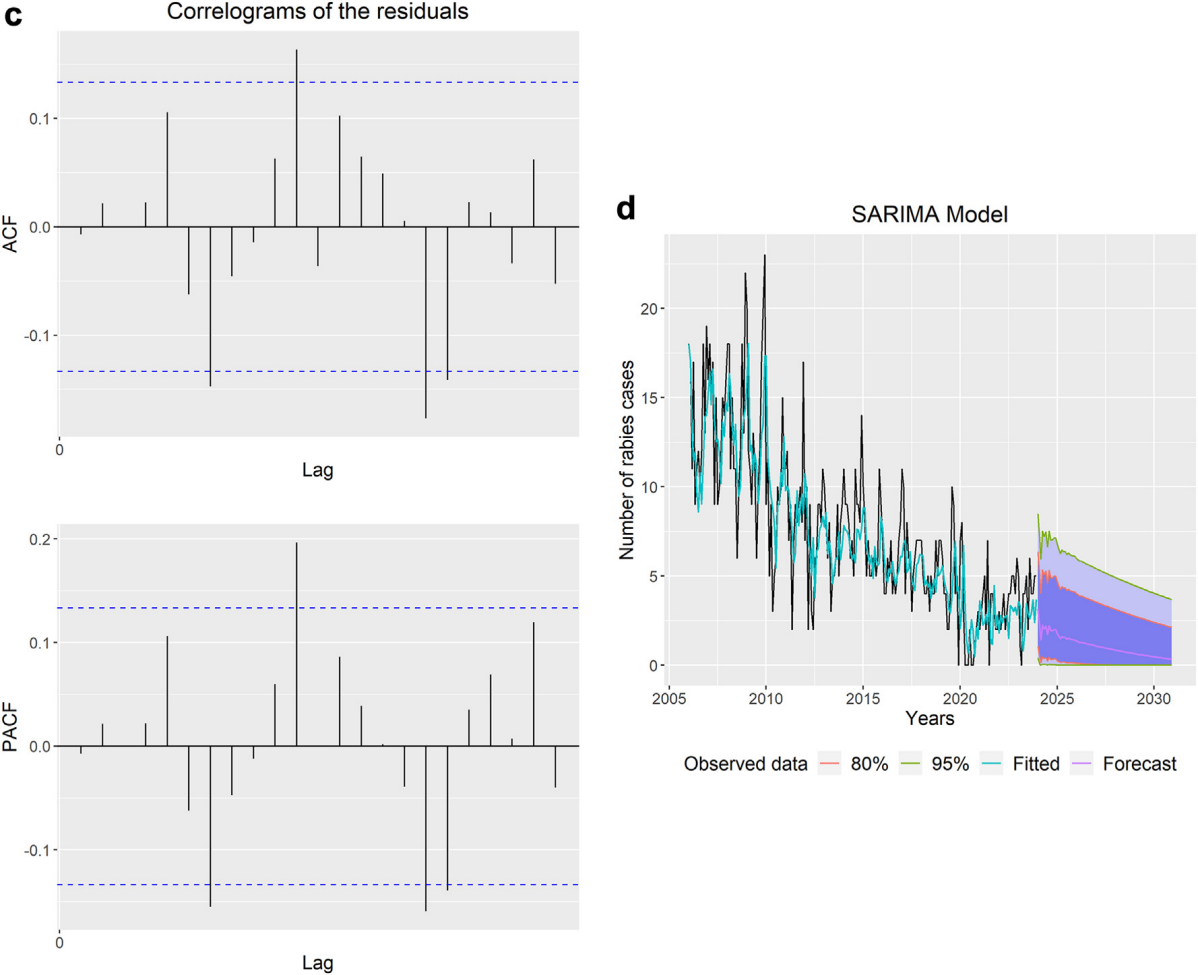
(a): Decomposition of the time series of rabies cases, (b): Correlograms of the time series of rabies cases, (c): Correlograms of the residuals of the SARIMA model, (d): Observed and predicted annual rabies cases using Seasonal Auto-Regressive Integrated Moving Average (SARIMA) model. The Y-axis represents the number of cases, and the X-axis represents the time (the number of years).

We found that the years 2006, 2007, and 2008 had the highest number of human rabies cases, with 167, 166, and 165 cases, respectively. On the other hand, the lowest occurrence of cases was observed in 2020, 2021, and 2022, with 26, 38, and 45 cases, respectively. Initially, there was a declining trend in cases from 2006 to 2013, followed by an increase in 2014, but the trend then resumed a downward trajectory. However, starting in 2015, the trend fluctuated, with periods of improvement and decline [Fig. 5a]. In recent years, starting from 2021, there has been a slight increase in rabies cases, rising from 38 in 2021 to 45 in 2023. When examining the monthly data, it was observed that rabies cases were most prevalent between November and February. The highest number of rabies cases occurred in December 2009 (n = 23), followed by December 2008 (n = 22), January 2009 (n = 20), November 2009 (n = 20), and December 2006 (n = 19), making them the top five months with the highest rabies incident rates.

The time series data representing rabies cases were analysed by decomposing it into three components: trend-cycle (T_t_), seasonal (S_t_), and error/residual (E_t_). To determine the suitable polynomials (p and P) and (q and Q) for the SARIMA model, we examined the autocorrelation function (ACF) and partial autocorrelation function (PACF) correlograms [Fig. 5b]. These correlograms assisted in specifying and identifying the suitable parameters for the SARIMA model.

Based on the results of significance tests and the analysis of residual correlograms using the ACF and PACF, the SARIMA (2,2,3) (1,0,0)_12_ model was estimated. The correlograms showed that all lag values fell within the confidence interval, confirming the chosen model [Fig. 5c].

We observed a decreasing trend with slight seasonality in human rabies cases by analysing the time-series frequency and utilising the observed number of rabies cases from January 2006 to December 2023. The SARIMA model demonstrated better predictive capabilities, as evidenced by the R^2^, RMSE, and MAE values of 94.15%, 2.67, and 2.04, respectively. This model is also relatively high-quality in BIC, AIC, and AICc, with 716.27, 692.70, and 693.25 values, respectively. The model identified a consistent downward trend between observed and predicted human rabies cases (Table 1). The model's projections suggest that Bangladesh is poised to make substantial progress towards achieving the ‘Zero by 30’ goal if the current trend of decreasing rabies cases continues [Fig. 5d].

**Table 1:** Prediction^a^ and summary of the SARIMA model using rabies cases from January 2024 to December 2030.

SARIMA (2,2,3) (1,0,0)_12_	January	February	March	April	May	June	July	August	September	October	November	December
2024	3	2	1	2	2	2	1	1	1	1	1	1
2025	1	1	1	1	1	1	1	1	1	1	1	1
2026	1	1	1	1	1	1	1	1	1	1	1	1
2027	1	1	1	1	0	0	0	0	0	0	0	0
2028	0	0	0	0	0	0	0	0	0	0	0	0
2029	0	0	0	0	0	0	0	0	0	0	0	0
2030	0	0	0	0	0	0	0	0	0	0	0	0
	RMSE^a^: 2.67		MAE^b^: 2.04		R^2c^ (%): 94.15		BIC^d^: 716.27		AIC^e^: 692.70		AICc^f^: 693.25	

[^a^RMSE, Root mean square error; ^b^MAE, Mean absolute error; ^c^R^2^, Coefficient of determination; ^d^BIC, Bayesian information criterion; ^e^AIC, Akaike information criterion; ^f^AICc, Akaike information criterion with correction].

a Rounded values.

In the count time-series generalized linear model (TSGLM), the impact of each variable is represented by the incidence risk ratio (IRR). According to this model, it was observed that with every unit increase in MDV, the relative risk of rabies cases decreased by 1% (IRR = 0.99 [95% CI 0.99–1.00], p = 0.099). Similarly, for each unit increase in ARV, the relative risk of rabies cases in Bangladesh decreased by 1% (IRR = 0.99 [95% CI 0.98–0.99], p < 0.001). The TSGLM model demonstrated better predictive capabilities, as evidenced by the AIC, BIC, RMSE, and R^2^, with the values of 740.42, 749.37, 5.06, and 74.83, respectively (Table 2).

**Table 2:** Risk ratio of mass dog vaccination (MDV) and anti-rabies vaccine (ARV) to rabies cases in Bangladesh using time-series generalized linear model methods.

	IRR^c^ (95% CI)	p-value
MDV	0.99 (0.99–1.00)	0.099
ARV	0.99 (0.98–0.99)	<0.001
AIC^a^: 740.42	RMSE^d^: 5.06	
BIC^b^: 749.37	R^2e^ (%): 74.83	

[^a^AIC, Akaike information criterion; ^b^BIC, Bayesian information criterion; ^c^IRR, Incidence risk ratio; ^d^RMSE, Root mean square error; ^e^R^2^, Coefficient of determination].

## Discussion

This integrative rabies control programme using the One Health approach demonstrates the viability and sustainability of such a programme in Bangladesh, providing a tangible example of achievement in eliminating dog-mediated human rabies deaths by 2030.

We employed time-series forecasting models using reported cases in the near past. There have been few, if any, detailed studies published on the forecasting of human rabies in Bangladesh. Infectious disease modeling is employed to comprehend the disease's dynamics better and assist with developing control strategies. Additionally, it reveals how dynamics and intervention tactics may alter as management is put in place.^42^^,^^43^ Based on the models, we identified a declining trend with slight seasonality in human rabies cases from January 2006 to December 2023. The SARIMA model demonstrated excellent predictive abilities, with R^2^, RMSE, and MAE values of 94.15%, 2.67, and 2.04, respectively. When comparing the observed and predicted human rabies cases, the model suggested a consistent decrease in number. Based on the model's projections, if this downward trend in human rabies cases continues, Bangladesh could make substantial progress towards achieving the ‘Zero by 30’ goal. Model predictions and surveillance studies with similar socioeconomic settings showed that dog vaccination could save human lives and prevent considerable human rabies exposure over a certain period.^44^ Our previous study on rabies in Bangladesh identified a trend of declining human rabies cases that can be explained by the increased number of dogs being vaccinated against rabies.^6^ Countries with 70% or higher vaccination coverage were classified as Phase III, and it was suggested that countries vaccinate 70% of the canine population for seven years to eliminate dog rabies.^1^^,^^45^^,^^46^ The frequent barriers to vaccination included a lack of awareness regarding the necessity of rabies vaccinations for dogs and ignorance about where to access the vaccine.^47^

Despite the strong political commitment to eliminate dog-mediated human rabies, allocating a budget for scaling up MDV in Bangladesh presents a challenge.^8^ Achieving the ‘Zero by 30’ goal requires significantly enhancing MDV rates, necessitating vital resources from national and international bodies to meet this target promptly. Dogs are the primary source of rabies exposure, but cats contribute significantly (12.11%) in Bangladesh, underscoring the importance of promoting vaccination after cat exposure.^5^ It has been demonstrated that rabies prevention strategies, such as vaccination, are influenced by disease prevalence and that a simple model with intervention responses can accurately depict the host dynamics and periodicity of the disease.^31^ Other factors can influence rabies cases, including literacy rate, GDP, awareness of rabies, access and availability of post-exposure vaccines, access to primary care, and contact related to sickness.^6^^,^^48^^,^^49^ However, reliable data on these parameters or proxy variables are still being determined. Therefore, in our study, we chose to include MDV and ARV in the model as the crucial determinants of the occurrence of human rabies cases. After MDV was introduced in Bangladesh in 2011, the pattern of human rabies cases altered quite quickly. The annual incidence of rabies in Bangladesh has consistently decreased as a result of the combined effects of a mass awareness campaign, PEP, and MDV.^6^

As per our analysis of rabies cases reported during 2011–2023, we found that rabies cases peaked between November and February. December 2009 had the highest number of cases (n = 23), followed by December 2008 (n = 22), January 2009 (n = 20), November 2009 (n = 20), and December 2006 (n = 19), making them the top five months with the highest incident rates. Corresponding trends have been observed in previous research in Bangladesh, where the lowest instances have been identified in June and the greatest in January, with peaks in December and January and then again in April. However, no association was observed between monthly temperature or rainfall and the number of animal bite cases.^50^ In contrast, most dog bite cases occur between the spring and summer months in developed countries.^51^ Such observations have been attributed to behavioral changes: more significant interaction between pets and children during the warmer months, with less parental monitoring, increasing the incidence of biting.^52^ A study in the African region showed that the highest human rabies exposures were reported in spring (April to June), followed by winter (January to March), with autumn (October to December) having the lowest incidence of human rabies exposure.^53^

Understanding the phylogenetic relationships of rabies viruses in different species, including wildlife, is crucial for rabies control efforts. This knowledge helps pinpoint potential virus reservoirs and assess the risk of spillover to domestic animals like dogs. While phylogenetic analysis may not determine vaccine strains for canine rabies, it aids in understanding transmission dynamics and identifying infection sources. Considering the broader context of rabies control, especially in neighboring countries where rabies control is not prioritised, including wildlife rabies and spillover risk, along with adopting a regional approach, is essential for sustainable elimination of dog-mediated rabies. The phylogenetic tree showed that the rabies viruses in Bangladesh are part of the Arctic/Arctic-like virus, and the N gene sequences from the rabies viruses in Bhutan and Bangladesh exhibit a significant link, indicating that they shared an ancestor before evolving into different strains. AAL2 spread into central Bangladesh 32.3 years ago (95% HPD 18.4–50.6 years) around 1978 (95% HPD range 1958–1991), where glycoprotein has three potential N-glycosylation sites influencing viral pathogenesis.^54^ Separate lineages were also discovered in other countries in this region, including Iran, Nepal, Pakistan, and Afghanistan.^9^^,^^54^ The rabies virus's diversity may have public health consequences in Bangladesh. Given the ease with which people can travel between countries, AAL2 most likely entered Bangladesh from India rather than Bhutan.

Our analysis shows that the estimated dog population in Bangladesh is 1,668,140, with an average density of 12.83 dogs/km^2^ and a human-to-dog ratio of 86.70. A prior study in Bangladesh revealed the estimated dog population was about 1.7 million, 14 dogs per km^2^, with a human-to-dog ratio of 120.^55^ The high ratio may be attributed to the abundance of edible waste on the streets, societal tolerance of stray dogs, and a lack of consistently implemented long-term birth control programmes.^56^ While Bangladesh has almost three times the benchmark number of dogs per km^2^ to become endemic for rabies, the threshold density for rabies persistence is only 4.5 dogs per km^2^.^55^^,^^57^ Bangladesh's dog population density is comparable to other Asian^57^ and African countries.^58^

From 2011 onward, we identified an increasing trend of ARV utilisation and MDV followed by a decreasing trend of human rabies cases in Bangladesh. After MDV was introduced in Bangladesh in 2011, the pattern of human rabies cases altered quite quickly.^6^^,^^8^ In recent years, human rabies cases have decreased by about 50%, with an annual reduction of approximately 12 cases, while the number of vaccinated dogs has increased by 3200 per year.^6^^,^^8^ The annual incidence of rabies in Bangladesh has consistently decreased due to the combined effects of a mass awareness campaign, PEP, and MDV. This accomplishment results from coordinated efforts by the Ministries of Health and Family Welfare, Fisheries and Livestock, Local Government, Rural Development & Cooperative, and Education. Together, they carried out rigorous MDV campaigns, offered PEP to animal bite victims, and increased awareness nationwide through ACSM. Working together across sectors is essential to achieving the Zero by 30 goals and is a shining example of what a One Health approach can accomplish.^17^ All 64 of Bangladesh's districts now have at least one facility that offers PEP and wound care to people whom animals have bitten.^6^ Additionally, Bangladesh created more than 300 sophisticated Animal Bite Management Centres at the national, district, and sub-district (Upazila) levels, in addition to preventing rabies at the source (the dogs). It is critical to guarantee that distant communities have access to medical treatment. The decision to visit one of the bite treatment centres is heavily influenced by travel time and accessible transportation options. They now ensure an ongoing supply of human rabies vaccine and immunoglobulin to deliver rabies post-exposure prophylaxis to over 400,000 bite sufferers annually.^17^ Over 250,000 individuals have received this care from trained nurses and doctors at no cost. The rise of MDV has confirmed the potential for successful rabies elimination strategies to result from a multi-sectoral, One Health strategy combining innovation, capacity-building, and broad implementation.^6^^,^^8^

Our findings demonstrated that some simple known interventions could help address crucial public health issues and how a genuine One Health approach should be used to control zoonotic diseases like rabies. Additional factors, such as increased awareness of rabies and availability of PEP for animal bite victims, along with MDV, may contribute to changes in the detection of human rabies cases in Bangladesh.^6^ MDV offers direct and indirect benefits in rabies control by spreading information about rabies, dog vaccines, and PEP among pet owners and communities. The direct benefits of MDV include controlling dog rabies by interrupting the transmission of RABV between dogs, while MDV indirectly contributes to rabies elimination by raising awareness of the disease, encouraging responsible pet ownership, meeting national public health goals, promoting first aid practices, personal hygiene, and other pet vaccinations. MDV also plays a role in preventing bites from pets and free-roaming dogs, supporting sterilisation and animal welfare, enhancing community health, and improving waste management practices. Furthermore, MDV can facilitate more robust relationships and trust between government agencies and the public through collaborative efforts to address rabies control. This heightened awareness increases the likelihood of seeking PEP treatment, establishing a positive correlation between MDV coverage and PEP utilisation. Overall, MDV saves lives and has broad implications for human and animal health through the One Health approach.

We recommend strengthening the existing rabies surveillance system in Bangladesh by utilising the state-of-the-art capabilities of the Central Disease Investigation Laboratory (CDIL) for rabies diagnosis through methods like direct fluorescent antibody test (DFAT), reverse transcription polymerase chain reaction (RT-PCR), and quantitative reverse transcription polymerase chain reaction (RT-qPCR); implementing rapid diagnostic tests (LFA) at Field Disease Investigation Laboratories (FDIL) for swift field-level diagnosis; establishing a dedicated human rabies diagnosis facility; introducing an active surveillance system; and conducting multicentric surveys. Nevertheless, obstacles such as cultural barriers, limited availability of antemortem diagnostic tools, and inadequacies in resources and infrastructure present challenges that must be overcome to improve the surveillance system and effectively manage rabies in Bangladesh. Also, ensuring the affordability, availability, and accessibility of human PEP, immunoglobulin, and the facilities providing these treatments is essential, requiring them to be accessible and cost-effective locally. We also recommend further research and advocacy to promote the adoption of alternative vaccination methods like oral rabies vaccines for both dogs and wildlife in the future. Furthermore, implementing a comprehensive waste management plan that includes efficient waste collection and disposal systems, waste segregation at source, recycling initiatives, and increased public awareness of responsible waste management practices in rabies-endemic countries like Bangladesh is necessary.^59^ This will help reduce the availability of food sources for free-roaming dogs, control stray dog populations, and minimise the risk of rabies transmission to humans, ultimately contributing to improved public health outcomes and rabies control efforts.

Like most other countries, Bangladesh was significantly impacted by the COVID-19 pandemic, especially during its initial wave, affecting rabies control activities such as PEP and MDV.^60^ Despite disruptions in vaccine supply chains that hindered rabies prevention services and reduced resources allocated to control efforts, Bangladesh exhibited resilience and determination in addressing these challenges.^17^ Through strong political commitment and unwavering will, the country navigated the complexities of global coordination and local vaccine delivery. Disruptions in vaccine supply chains have hindered rabies prevention services, while lockdown measures indirectly reduced reported dog bite cases due to decreased human–dog interactions. Bangladesh's proactive approach to maintaining effective rabies control programs, including MDV campaigns and timely provision of PEP services amidst the pandemic, reflects a positive and proactive attitude towards overcoming obstacles and safeguarding public health.

Our investigations had some limitations. Our study used a comprehensive dataset from 2011 to 2023 to estimate the dog population. We initially employed manual data collection methods with paper and MS Excel but switched to KoBo Toolbox mobile apps in 2017, applying the Lincoln-Petersen formula with Chapman's correction. This transition may have affected the accuracy of our estimates and, hence, the model prediction in the study. Notwithstanding, our estimation remains robust and supported by the comprehensive dataset. We focused on human rabies cases identified at public hospital facilities (NRPCC/DRPCCs). While we acknowledge that some cases may have been overlooked, particularly those treated at private hospitals or by traditional healers, we estimate that only a tiny proportion of rabies deaths were not captured by the government's primary data collection mechanisms due to advancements in education and awareness.

Patients relying on relatives for historical recollections may introduce recall bias. We mitigated this by directly consulting some relatives via phone. Despite potential limitations, our findings maintain an acceptable level of accuracy and provide valuable insights into rabies control in Bangladesh.

MDV programmes have shown positive outcomes, with increased vaccination and decreased human rabies cases in Bangladesh from 2011 to 2023. The trend in human rabies cases has fluctuated but could be eliminated if the decreasing trend continues. A declining trend in predicted and observed human rabies cases has been identified, suggesting that Bangladesh is poised to make substantial progress towards achieving the ‘Zero by 30’ goal, provided the current trajectory continues. Increasing MDV and ARV led to a decline in the relative risk of human rabies cases. The rabies virus circulating in Bangladesh belongs to the Arctic/Arctic-like virus, and experts should continue coordinating vaccination efforts. Our findings demonstrate that successful MDV programmes operations are feasible in Bangladesh and can eliminate dog-mediated human rabies across broad geographic areas. Our research findings have the potential to inform rabies control strategies in Bangladesh and countries with similar socio-economic settings, contributing to the global effort to achieve the ‘Zero by 30’ goal.

## Contributors

Conceptualisation, S.G., M.N.H., and N.H.; Methodology, S.G., M.N.H., N.D., D.J., and N.H.; Data Analysis, M.N.H., N.D., D.J., M.J.U., and S.G.; Field Investigation, M.K.I., M.M.R., N.M., M.K., M.F.R., S.K., S.M.U., M.R.A.S., M.S.R., and A. A. J.; Data Processing, M.N.H., N.D., S.G. and H.S.M.; Writing—Original Draft, S.G., M.N.H., N.H. and S.C.; Supervision, T.S.S., M.N.I., S.M.G.K., B.A., and U.R.S.

## Data sharing statement

Data supporting the findings of this study can be found in the publication and its Supplementary Information files.

## Editor note

The Lancet Group takes a neutral position with respect to territorial claims in published maps and institutional affiliations.

## Declaration of interests

The authors declared that they have no competing interests.
