# Supplementary material for: Rabies control in Bangladesh and prediction of human rabies cases by 2030: a One Health approach
Source: Lancet Reg Health Southeast Asia. 2024 Jul 23;27:100452. doi: 10.1016/j.lansea.2024.100452 (PMC11321326; doi:10.1016/j.lansea.2024.100452)
Supplement: Supplementary File 1 [file mmc1.docx]

**Rabies control in Bangladesh and prediction of human rabies cases by 2030: a One Health approach**

*Sumon Ghosh^ab*^, Mohammad Nayeem Hasan^c^, Nirmalendu Deb Nath^d^, Najmul Haider^e^, Daleniece Higgins Jones^a^, Md. Kamrul Islam^b^, M. Mujibur Rahaman^b^, Hasan Sayedul Mursalin^b^, Nadim Mahmud^b^, Md. Kamruzzaman^b^, Md. Fazlay Rabby^b^, Shotabdi Kar^b^, Sayed Mohammed Ullah^b^, Md. Rashed Ali Shah^b^, Afsana Akter Jahan^b^, Md. Sohel Rana^f^, Sukanta Chowdhury^g^, Md. Jamal Uddin^c^, Thankam S. Sunil^a^, Be-Nazir Ahmed^b^, Umme Ruman Siddiqui^b^, S. M. Golam Kaisar^b^ and Md. Nazmul Islam^b^*

^a^ Department of Public Health, University of Tennessee, Knoxville, USA

^b^ Disease Control Unit, Communicable Disease Control, Directorate General of Health Services, Ministry of Health and Family Welfare, Bangladesh

^c^ Department of Statistics, Shahjalal University of Science and Technology, Sylhet, Bangladesh

^d^ Department of Biomedical and Diagnostic Sciences, University of Tennessee, Knoxville, USA

^e^ School of Life Sciences, Keele University, Staffordshire, the United Kingdom, ST5 5BG

^f^ Department of Livestock Services, Ministry of Fisheries and Livestock, Dhaka, Bangladesh

^g^ International Centre for Diarrhoeal Disease Research (icddr,b), Dhaka, Bangladesh

*Corresponding author. 382 HPER, 1914 Andy Holt Ave., Knoxville, TN 37996, USA

E-mail address: [sumon.ghoshbd@gmail.com](mailto:sumon.ghoshbd@gmail.com) (S. Ghosh)


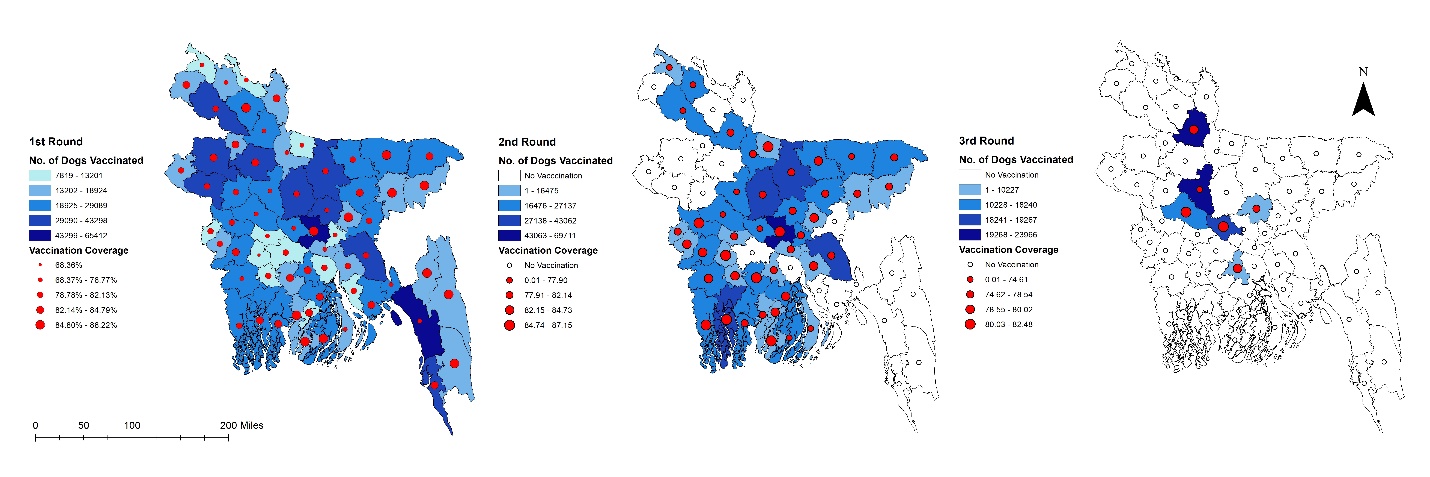


Supplementary Fig. 1: **Scaling up mass dog vaccination (MDV) with vaccination coverage (%) in different districts of Bangladesh, 2011-2022**. Left: Showing the number of dogs vaccinated (blue shadow) with coverage (red circles) during the first round of MDV. Middle: Showing the number of dogs vaccinated (blue shadow) with coverage (red circles) during the second round of MDV. Right: Showing the number of dogs vaccinated (blue shadow) with coverage (red circles) during the third round of MDV.

[Thirty-five districts (Narsingdi, Narayanganj, Munshiganj, Jamalpur, Sherpur, Mymensingh, Kishoreganj, Netrokona, Tangail, Sylhet, Sunamganj, Moulvibazar, Habiganj, Dinajpur, Panchagarh, Gopalganj, Rajbari, Satkhira, Bagerhat, Jessore, Khulna, Meherpur, Kushtia, Chuadanga, Narail, Jhenaidah, Magura, Barisal, Jhalokathi, Patuakhali, Pirojpur, Barguna, Bhola, Chandpur, and Cumilla) already having completed their second round of vaccination, while eight districts (Gaibandha, Sirajganj, Pabna, Gazipur, Manikganj, Madaripur, Nilphamari, and Dhaka) completed their third round of vaccination.]


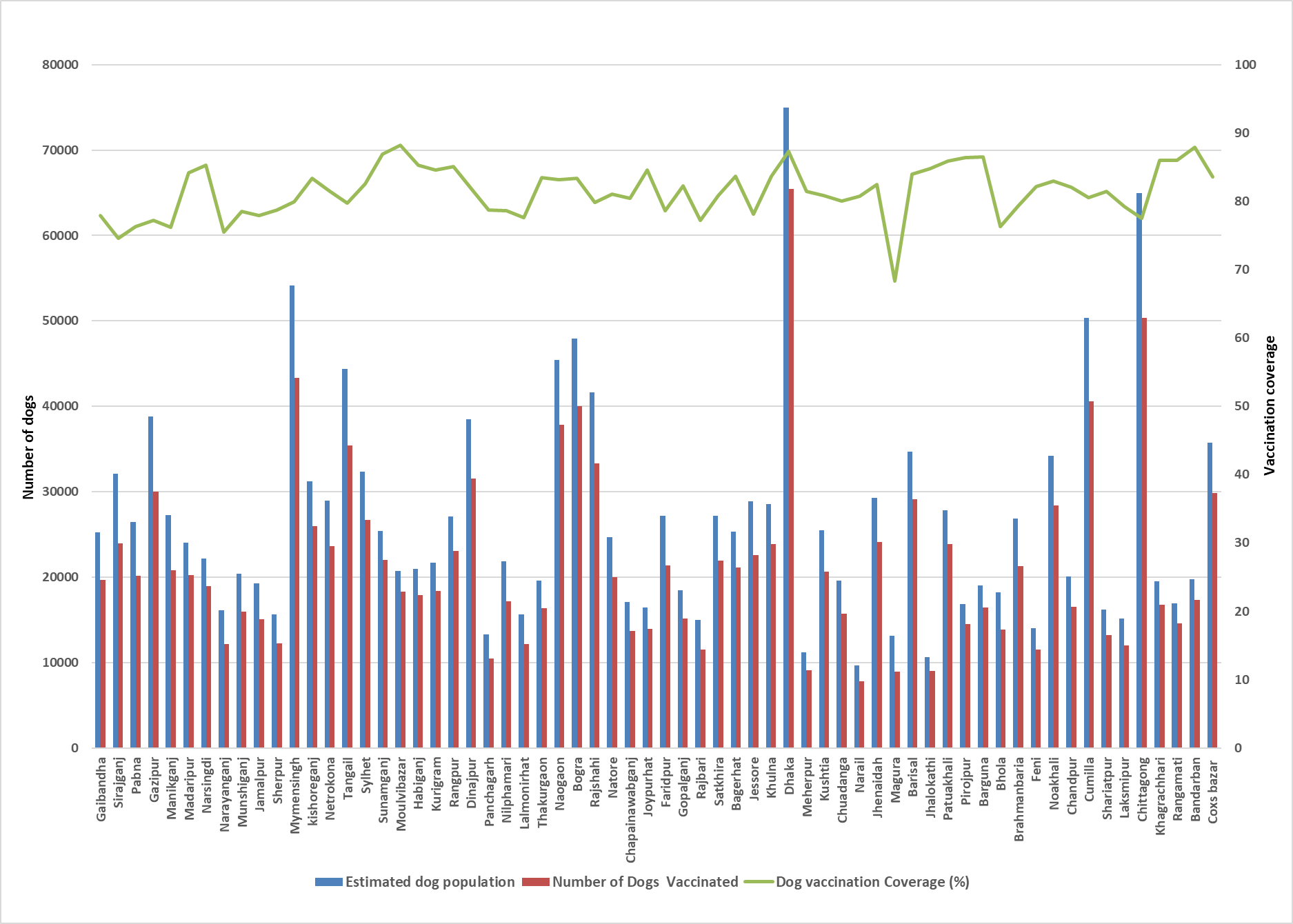


**Supplementary Fig. 2: District-wise estimated dog population (blue bars), number of dogs vaccinated (red bars), and vaccination coverage (green line) in Bangladesh, 2011-2022.**


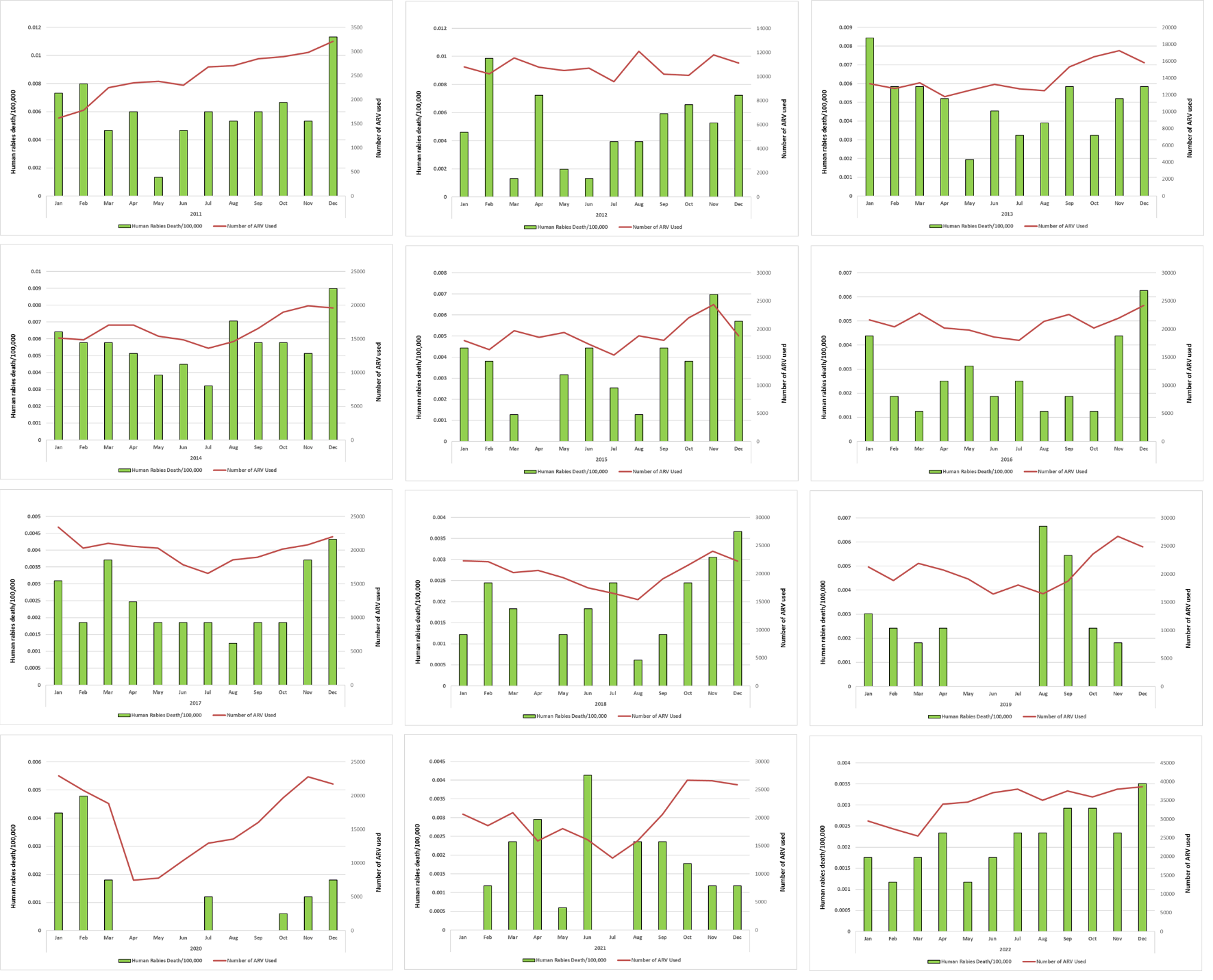


**Supplementary Fig. 3 (a): Graph of month-wise human rabies deaths (green bar) and number of anti-rabies vaccine utilisation (solid line) reported at the National Rabies Prevention and Control Centres (NRPCC) of the Infectious Disease Hospital (IDH), Dhaka, Bangladesh, 2011–2022.**


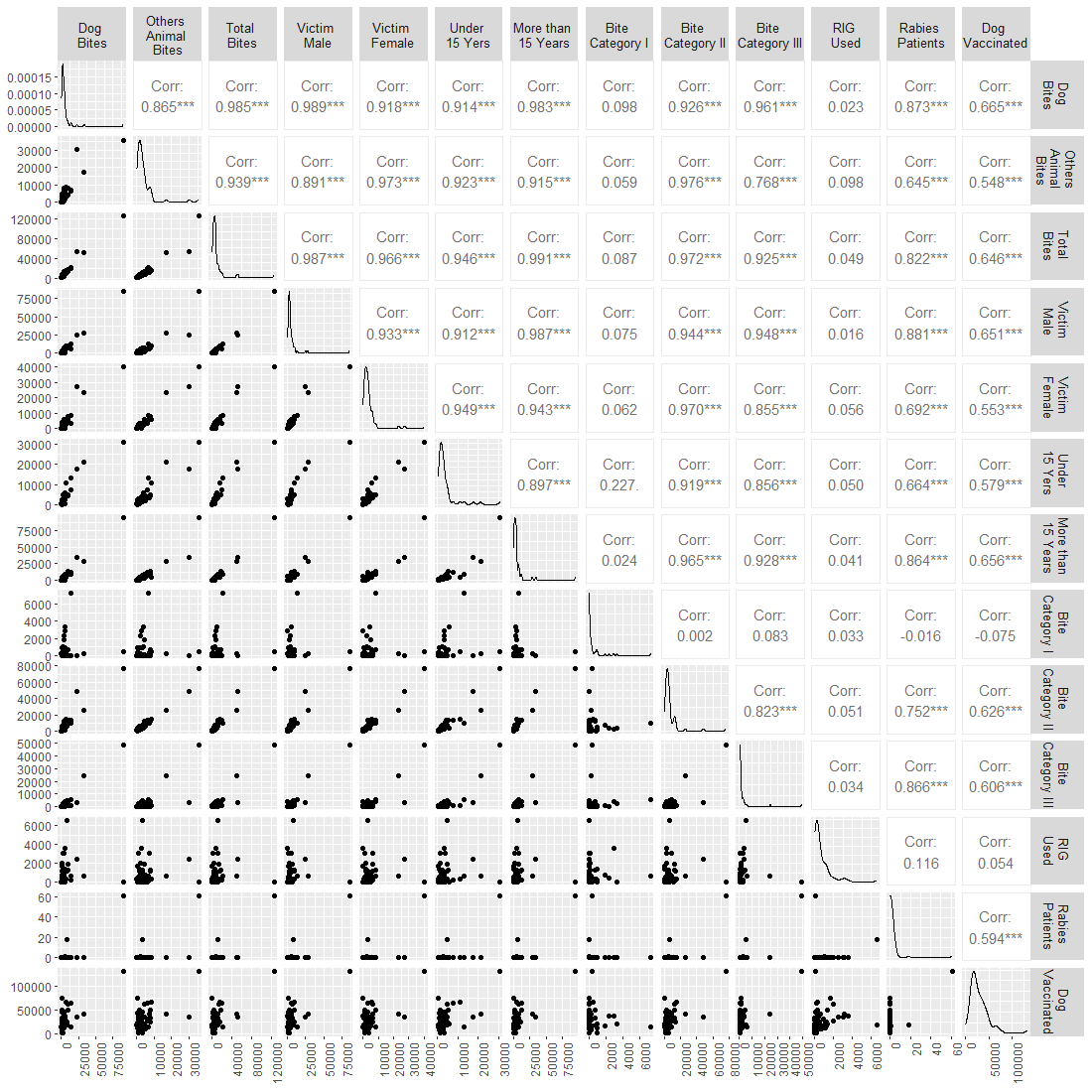


**Supplementary Fig. 3 (b): Spearman rank correlation coefficients between various factors for confirmed human rabies cases**

[The Spearman rank correlation coefficients between key rabies patient characteristics and ARV variables point to a statistically significant association between these two variables. Dog bites (r = 0.865, p<0.05), other animal bites (r = 0.909, p<0.05), and total bites (r = 0.996, p<0.05) all show a strong positive correlation with the ARV.]


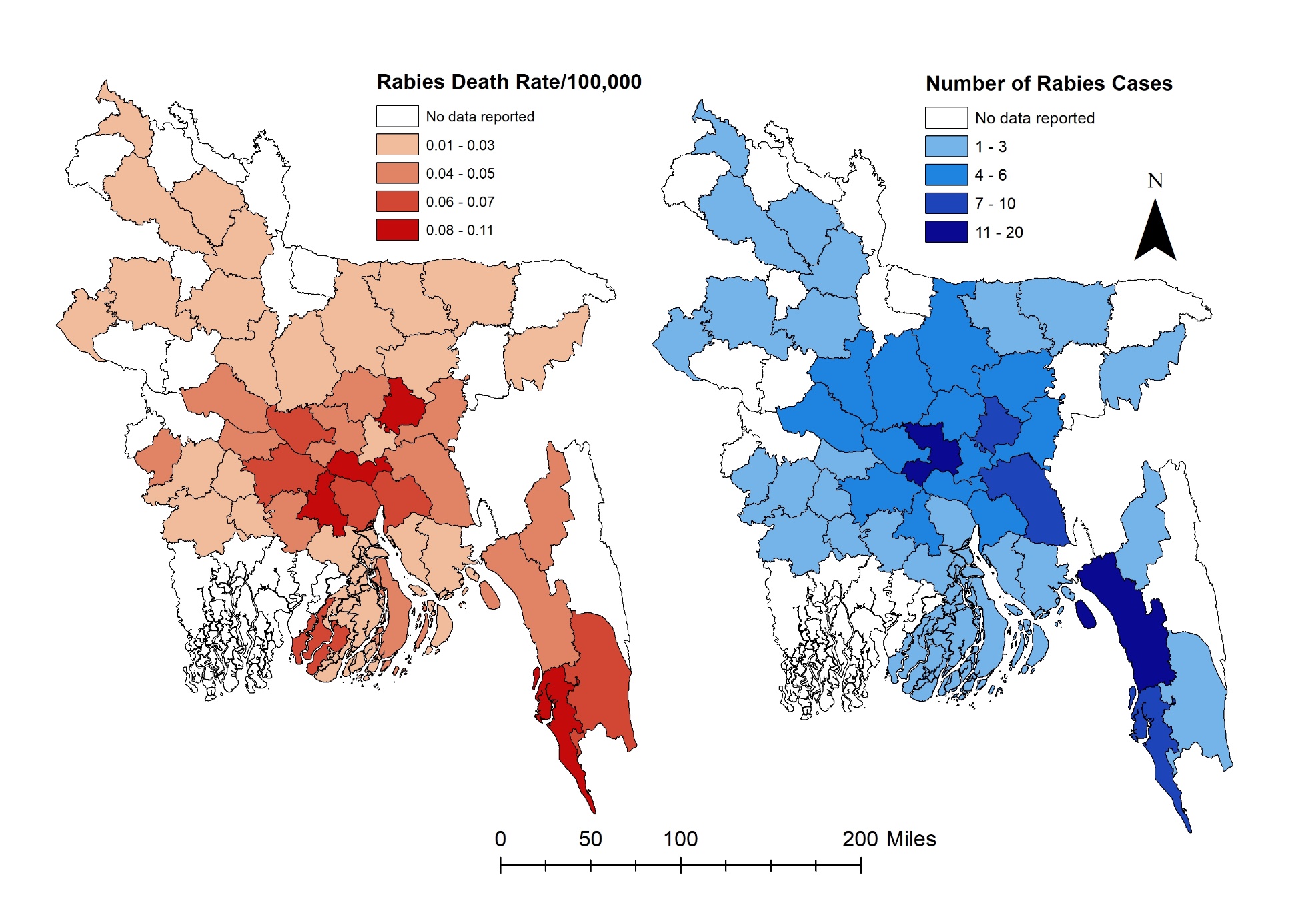


**Supplementary Fig. 4 (a): Distribution of human death rate (cases per 100,000 populations) (left) and rabies cases (right) in different districts of Bangladesh reported at the National Rabies Prevention and Control Centres (NRPCC) of the Infectious Disease Hospital (IDH), Dhaka, Bangladesh, 2019–2022.**


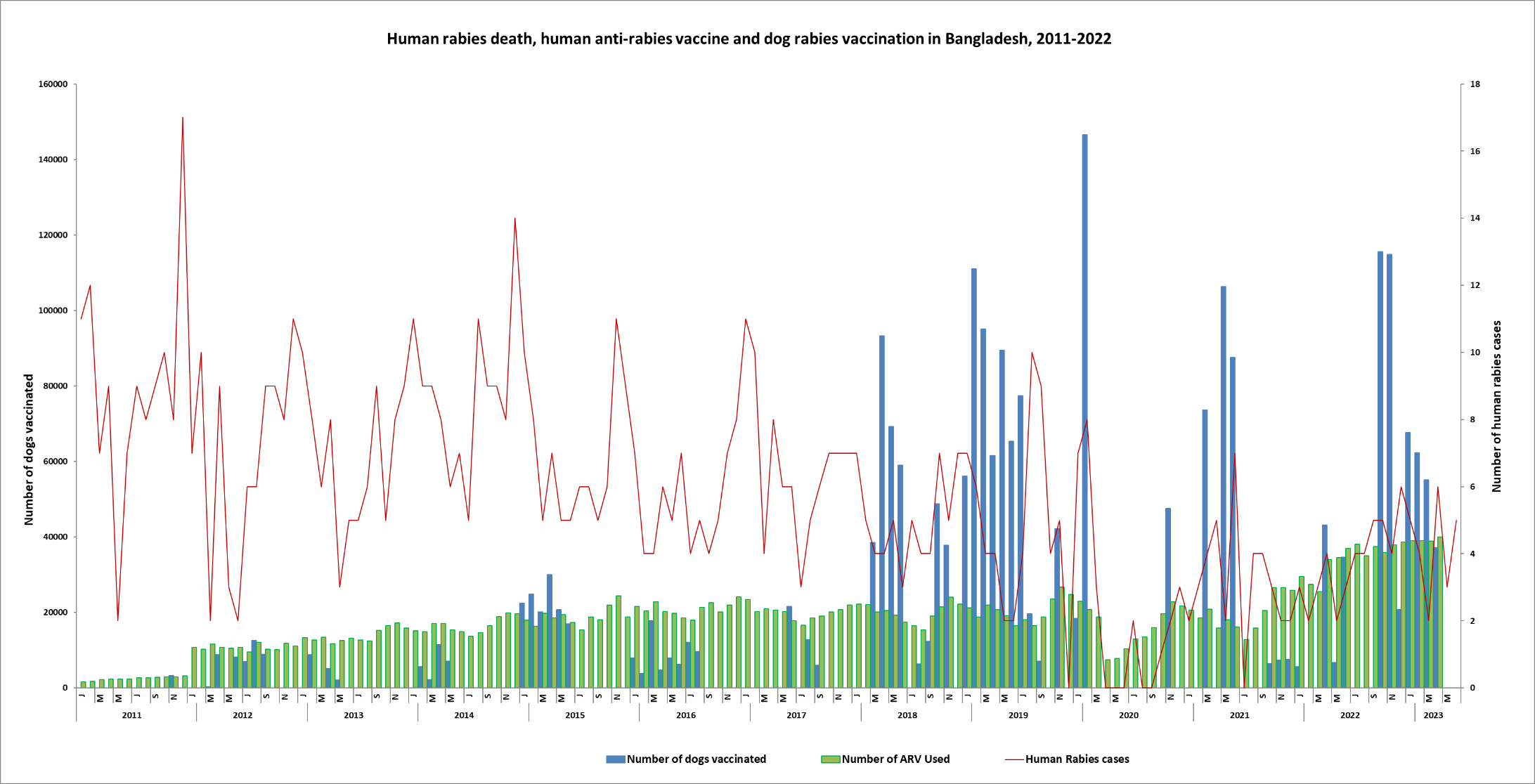


**Supplementary Fig. 4 (b): Month-wise trends of human rabies deaths with respect to anti-rabies vaccine and mass dog vaccination in Bangladesh, 2011-2022.**

**MDV:**

**Methods:**

- 1. **Advance planning**

Preliminary planning meeting have organized with key stakeholders and rabies experts from health, livestock, and local government to agree on project objectives organize logistics and assign responsibilities. An initial assessment have made to gather baseline information on the following parameters (in order to better plan the mass vaccination and estimate the resources required)

- Number of households
- Dog population size
- Maps and ward boundaries
- Before conduction of MDV the following events are organized:
  - Preparatory meeting
  - Training of Animal Control Staff (ACS)
  - Micro-planning
  1. **Communications**

Before the mass vaccination was conducted, it was necessary to sensitize the local community so they were aware of the objectives of the program. This could enable greater vaccination coverage to be achieved. Communications activities were initiated two days prior to the start of mass vaccination and were continued throughout the entire duration of mass vaccination.

- 1. **Key messages**

The key messages chosen for the Communications program were:

- The Government of Bangladesh is starting a rabies elimination programme in the country
- Rabies in humans can be eliminated through vaccination of dogs
- Government vaccination teams will try to vaccinate all dogs against rabies
- Please cooperate and assist vaccination teams in finding dogs
- Be kind to dogs and handle them with care

**d. Communication logistics**

- Banners and posters –to use before and during MDV program
- Leaflets and brochures – to distribute before and during MDV campaign
- “Miking” to conduct before and during MDV campaign (in Bengali)
- Launching event – mass parade in MDV area

**e. Catching and handling dogs for vaccination**

All dogs were vaccinated regardless of ownership status (i.e. regardless of whether they were owned ‘pet’ dogs, or community-owned dogs, or un-owned ‘stray’ dogs). Puppies under one month of age and dogs deemed to be sick were excluded from vaccination.

It was important to minimize stress to the dogs and handle them with kindness during MDV. A fearful dog was very difficult to catch and may be aggressive. Thus, minimizing stress to the dogs also meant that dogs were easier to catch and handle during first and any subsequent vaccination campaigns.

**f. Vaccine storage and transport**

Vaccines were stored in the municipality health office/UH&FPO’s office, where the refrigerator was available and continuous power supply was ensured. Enough vials of vaccine were provided to each MDV team in the very early morning of the vaccination day according to local demand. To avoid system loss, opened vials were used first. During transportation to the field sites and on the vaccination day, vaccines were stored in good quality cool boxes with ice packs. Vaccination team were responsible for managing the vaccine cold chain (between 2° and 8°C). Overnight, vaccines were safely returned to refrigerated storage. Used and unused vials were counted in and out to each team on a daily basis. All used needles and syringes were placed in disposal bags in the field and each day transferred to a safety box at the municipality/upazila health office for safe disposal.

**g. Distribution of vaccination team:**

Generally, two/three teams worked in each union/ward for a total of 4-6 days. However, it may vary depending on the burden of dog in the particular areas, size of the area and availability of the resource.

**h. Injection protocol**

Vaccination was performed by the trained animal vaccinator(s) in the team, who stood behind the dog while vaccinating. A new needle and syringe were used for every dog receiving a vaccine.

Dogs being held by their owner or Dog Handler were vaccinated subcutaneously (this was less painful hence minimizing the risk of dog bites) using one of the following sites:

- Neck – recommended site as the skin is loose and easy to lift. The skin is held and the needle inserted either side of the midline above the vertebra, parallel to the skin fold, with care to avoid blood vessels.
- Ribs –often the most convenient and safest area.
- Skin flap on the flank –alternative site due to the loose skin.

Dogs in nets were generally vaccinated intramuscularly for a better immune response. The recommended site for intramuscular was the hind leg, carefully avoiding the Ischiatic nerve and leg bones which could be damaged during injection.

**i. Marking dogs**

It was important that dogs were given a mark to indicate that they had been vaccinated. This enabled a post-vaccination survey to estimate vaccination coverage. In our MDV campaign, every dog vaccinated was given a mark of a red collar or yellow paint, or both. Collars were not put on puppies, as there was a risk of strangulation if they were not removed as the puppy grew. Dogs caught using nets were sprayed with paint through the net, and generally did not receive collars. It was not advisable to give dogs a permanent mark during vaccination as this required general anaesthesia (for tattooing or ear notching), and the mark may have lasted longer than the conferred immunity. During marking, dog masks were used to avoid accidental bites and belts depending upon feasibility.

**j. Record-keeping**

Information on all dogs was recorded, including those that were not vaccinated by the team (due to escaping or being deemed too young or sick), or those that are vaccinated but not marked (due to escaping).

**Post vaccination dog survey:**

During the microplanning, vaccination teams were provided with direction regarding the location and route within the designated area. Ward maps from the local municipality were utilized to guide teams during the vaccination campaign. After the initial cycle of vaccinations in each ward, a surveyor traveled around the ward using various modes of transportation - motorbike, bicycle, or walking - while navigating with either a paper map or smartphone app to cover every street within the ward boundaries. Post-vaccination survey routes were established based on the directions given during microplanning. During the survey, the surveyor traversed through the area in a zig-zag pattern to cover the entire length and width of the ward without bias for specific areas^1^. They moved at a consistent pace to reduce the likelihood of encountering the same dog multiple times, ensuring thorough coverage of the area. Every street was carefully searched, with each observed dog recorded on the survey worksheet as either vaccinated (indicated by paint spray) or not vaccinated.

**Factor Analysis:**

For the purpose of factor analysis, we opted for the 'principal components' method as our chosen solution approach^2,3^. The count of factors resulting from the factor analysis denotes the potential sources of variance present within the data. These factors are ranked based on their significance. The foremost factor or principal component holds the highest eigenvalue, signifying the most pivotal contributor to the variance observed in the data. Conversely, the final factor represents the least influential process contributing to the overall chemical variance. The factor loadings are interpreted as correlation coefficients, elucidating the relationship between the variables and the factors. Through this approach, we extract a limited number of factors that generally encapsulate a similar amount of information as the original dataset. The observed variables are mathematically represented as linear combinations of these underlying factors, inclusive of error terms, illustrated as follows:

*Z_ij_* =*a*_1_*f*_1_*_j_* +*a*_2_ *f*_2_*_j_* +....+*a_m_f_mj_* +*e_ij_*

*Where
Z_ij_= Measured variable, a_i_= ith Factor loading, f_ij_= Factor score, e_ij_= Error term
i=1, 2, 3,…m, j=1, 2, 3,…p*

Cluster analysis, initially introduced by Tryon (1958), is a multivariate methodology designed to group cases together when their group memberships are not predetermined^4^. Essentially, it aims to categorize individuals or objects into unknown clusters, characterized by relatively consistent internal properties and notable differences between clusters, all based on a defined set of variables. These resulting groups are referred to as clusters. In the realm of biology, cluster analysis has long been employed in the field of taxonomy, where organisms are categorized into arbitrary groups based on shared characteristics, progressing from broader categories like kingdom down to more specific ones such as phylum, class, and so forth.

We utilized Hierarchical clustering method, which involves a sequence of either merging or dividing actions^5^. Notably, in the initial stages of this approach, there's no need to predefine the number of clusters. Hierarchical clustering constructs a cluster hierarchy, and the results are visually depicted through a Dendrogram. In this representation, each step of hierarchical clustering is portrayed as a fusion of two branches, progressively merging into a single branch, symbolizing the clusters derived at each stage of the process.

**Supplementary Table 1: Rotated factors for the animal bite patients reported at the National Rabies Prevention and Control Centre (NRPCC) and District Rabies Prevention and Control Centres (DRPCCs) of Bangladesh, 2019–2022 (Extraction method**)**

|  | PC1 | PC2 | PC3 |
| --- | --- | --- | --- |
| Dog Bites | 0.319 | -0.007 | -0.040 |
| Other Animal Bites | 0.300 | -0.051 | 0.022 |
| Total Bites | 0.322 | -0.022 | -0.020 |
| Male Victim | 0.320 | 0.011 | -0.043 |
| Female Victim | 0.309 | -0.043 | -0.024 |
| Victim Under 15 Years | 0.303 | -0.188 | -0.058 |
| Victim Equal or More than 15 Years | 0.320 | 0.051 | -0.011 |
| Bite Category I | 0.024 | -0.918 | -0.158 |
| Bite Category I | 0.312 | 0.041 | -0.005 |
| Bite Category III | 0.302 | 0.001 | -0.025 |
| RIG Used | 0.020 | -0.195 | 0.973 |
| Rabies Patients | 0.272 | 0.125 | 0.108 |
| Dog Vaccinated | 0.220 | 0.243 | 0.086 |
| Eigenvalue | 9.59 | 1.08 | 1.01 |
| Variance Explained (%) | 73.74 | 8.32 | 7.74 |
| Cumulative Variance (%) | 73.74 | 82.06 | 89.80 |

** Three components extracted from the matrix; Extraction method = Principal component analysis; Loadings greater than 0.3 are in bold.

[Principal component analysis (PCA) has extracted three significant PCs with eigenvalues > 1, which explain about 90% of the total variance in the data set of rabies characteristics of Bangladesh. PC1, PC2 and PC3 account for 73.74%, 8.32% and 7.74% of the total variance, respectively. CA has indicated five clusters or groups. Group A, Group B, Group C, Group D and Group E, respectively, consists of 10%, 20%, 15%, 25% and 30% of the total sample points.]

| 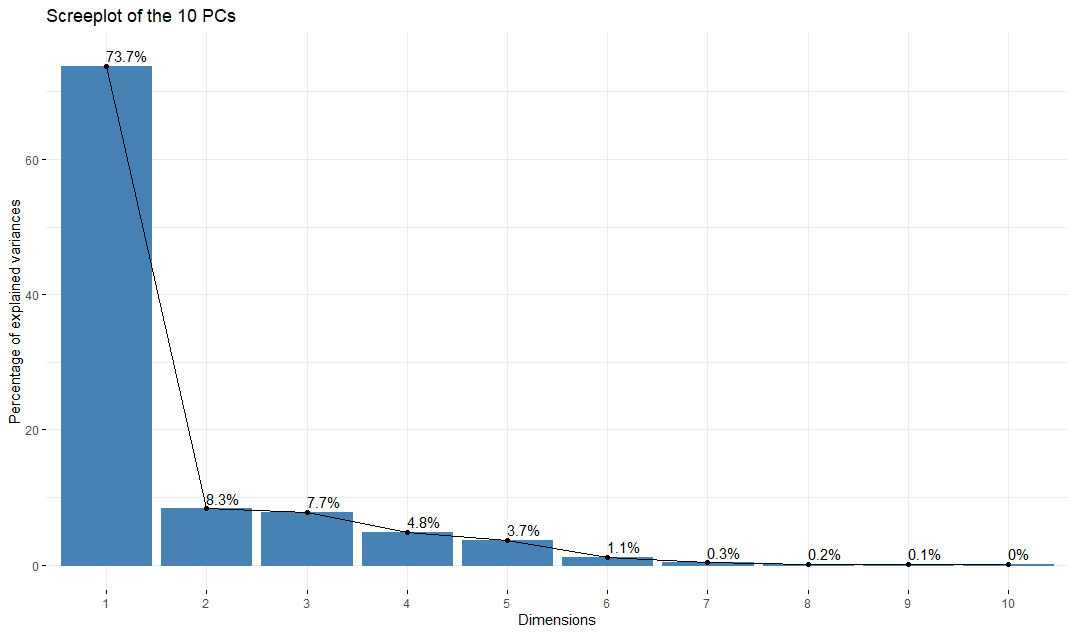 |
| --- |
| **Supplementary Fig. 5: Scree plot with % of explained variance** |

| 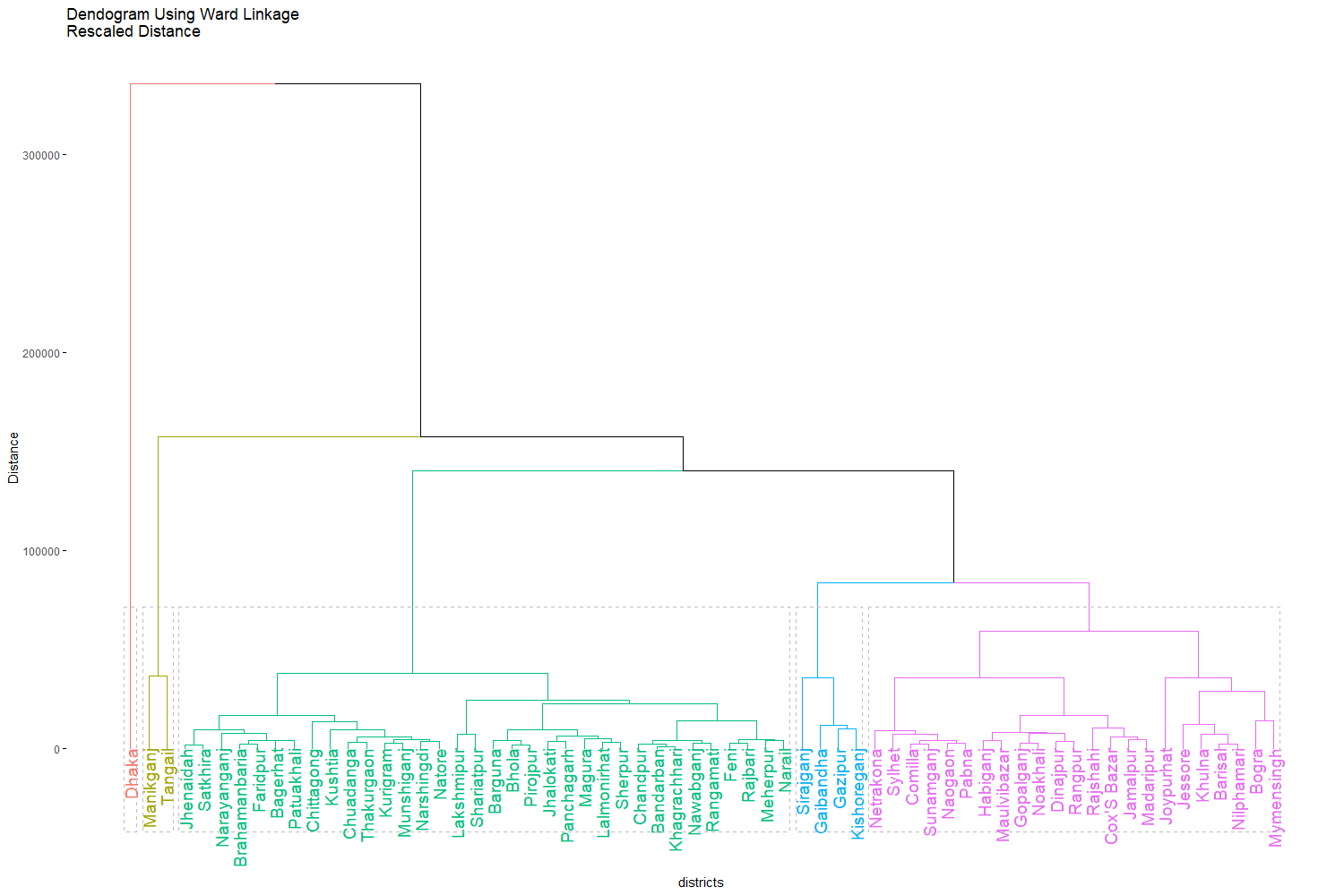 |
| --- |
| **Supplementary Fig. 6: Dendrogram of Cluster Analysis of District Parameters for the animal bite patients reported at the reported at the National Rabies Prevention and Control Centre (NRPCC) and District Rabies Prevention and Control Centres (DRPCCs) of Bangladesh, 2019–2022** |

**Supplementary Table 2: Cluster Groups and their Members (Districts) for the animal bite patients reported at the reported at the National Rabies Prevention and Control Centre (NRPCC) and District Rabies Prevention and Control Centres (DRPCCs) of Bangladesh, 2019–2022**

| Group | Members (Districts) | N (%) |
| --- | --- | --- |
| A | Dhaka | 2 (10) |
| B | Munshiganj, Tangail | 4 (20) |
| C | Gaibandha, Gazipur, Kishoreganj, Sirajganj | 3 (15) |
| D | Bagerhat, Chittagong, Jhalokati, Jhenaidah, Khagrachhari, Kurigram, Kushtia, Lakshmipur, Lalmonirhat, Magura, Munshiganj, Narail, Narayanganj, Natore, Nawabganj, Netrakona, Nilphamari, Noakhali, Pabna, Panchagarh, Patuakhali, Pirojpur, Rajbari, Rajshahi, Rangamati, Rangpur, Satkhira, Shariatpur, Sherpur, Sirajganj, Sunamganj, Sylhet, Tangail, Thakurgaon | 5 (25) |
| E | Bagerhat, Bandarban, Barguna, Barisal, Bhola, Bogra, Brahamanbaria, Chandpur, Chittagong, Chuadanga, Comilla, Cox's Bazar, Dhaka, Dinajpur, Faridpur, Feni, Gaibandha, Gazipur, Gopalganj, Habiganj, Jamalpur, Jessore, Jhalokati, Jhenaidah, Joypurhat, Khagrachhari, Khulna, Kishoreganj, Kurigram, Kushtia, Lakshmipur, Lalmonirhat, Madaripur, Magura, Manikganj, Maulvibazar, Meherpur, Munshiganj, Mymensingh, Naogaon, Narail, Narayanganj, Narshingdi, Natore, Nawabganj, Netrakona, Nilphamari, Noakhali, Pabna, Panchagarh, Patuakhali, Pirojpur, Rajbari, Rajshahi, Rangamati, Rangpur, Satkhira, Shariatpur, Sherpur, Sirajganj, Sunamganj, Sylhet, Tangail, Thakurgaon | 6 (30) |

**Supplementary Table 3: Association with age category and various factors of rabies patients reported at the reported at the National Rabies Prevention and Control Centre (NRPCC) and District Rabies Prevention and Control Centres (DRPCCs) of Bangladesh, 2019–2022**

|  | Age Category | |  | P-value |
| --- | --- | --- | --- | --- |
|  | ≤15 | >15 | Total |  |
| Data Source |  |  |  |  |
| BITID | 10 (43.48) | 13 (56.52) | 23 (13.22) | 0.258 |
| IDH | 46 (33.17) | 97 (67.83) | 143 (82.18) |  |
| Others | 1 (12.50) | 7 (87.50) | 8 (4.60) |  |
| Case Year |  |  |  |  |
| 2019-2020 | 24 (28.92) | 59 (71.08) | 83 (47.70) | 0.384 |
| 2021-2023 | 33 (36.26) | 58 (63.74) | 91 (52.30) |  |
| Sex |  |  |  |  |
| Male | 46 (35.66) | 83 (64.34) | 129 (74.14) | 0.232 |
| Female | 11 (24.44) | 34 (75.56) | 45 (25.86) |  |
| Residence |  |  |  |  |
| Rural | 54 (32.73) | 111 (67.27) | 165 (94.83) | 0.999 |
| Urban | 3 (33.33) | 6 (66.67) | 9 (5.17) |  |
| Vaccination |  |  |  |  |
| Present | 5 (100.00) | 0 (0.00) | 5 (2.87) | 0.006 |
| Absent | 52 (30.77) | 117 (69.23) | 169 (97.13) |  |
| Bites |  |  |  |  |
| Cat | 2 (20.00) | 8 (80.00) | 10 (5.75) | 0.416 |
| Dog | 46 (35.38) | 84 (64.62) | 130 (74.71) |  |
| Others | 9 (26.47) | 25 (73.53) | 34 (19.54) |  |
| Total | 57 (32.76) | 117 (67.24) |  |  |

Given that the outcome variable comprises binary repeated entries categorized by age groups, we employed a logistic mixed effects model (LMM). The rationale behind this choice lies in the LMM's ability to effectively handle repeated measurements within the same subject by incorporating subject-specific random effects, thus accounting for latent individual characteristics. Additionally, we employed a linear combination of predictor variables to model the log odds of the outcomes, particularly useful when data exhibit clustering or involve a combination of fixed and random effects. Initially, a comprehensive model was constructed, encompassing the chosen predictor variables. Subsequently, we employed a stepwise approach involving both backward and forward elimination techniques to derive the ultimate model. In the backward method, we progressively excluded the least statistically significant variable at each step, while in the forward method, we successively included highly significant variables. To validate the correct specification of the final model, we iteratively repeated the elimination procedure, manually incorporating all potential risk factors present in the initial comprehensive model.

**Supplementary Table 4: Parameter estimates from the best fitting Model**

| Fixed effect | OR | Lower CL | Upper CL | P-value |
| --- | --- | --- | --- | --- |
| Case Year | 0.90 | 0.90 | 0.91 | <0.001 |
| Gender |  |  |  |  |
| Female | 1.60 | 0.73 | 3.50 | 0.243 |
| Male | Reference |  |  |  |
| Residence |  |  |  |  |
| Urban | 0.69 | 0.16 | 3.01 | 0.624 |
| Rural | Reference |  |  |  |
| Bites |  |  |  |  |
| Others | 0.78 | 0.13 | 4.50 | 0.778 |
| Dog | 0.57 | 0.11 | 2.92 | 0.496 |
| Cat | Reference |  |  |  |
|  |  |  |  |  |

The data presented in Supplementary Table 4 demonstrate a decreased odds (OR = 0.90, 95% CI = 0.90, 0.91) of rabies occurrence among patients aged over 15 years when compared to those aged 15 years or younger, across the four observed time periods. Furthermore, our analysis revealed an elevated odds (OR = 1.60, 95% CI = 0.73, 3.50) of rabies infection among female patients aged over 15 years in comparison to their male counterparts aged 15 years or younger. These reduced odds were similarly associated with patients residing in urban areas, those affected by bites from animals other than dogs and cats, and those bitten by dogs, all within the greater than 15 years age group, as opposed to their rural counterparts aged 15 years or younger who were bitten by cats.

**Supplementary Table 5: The Accession Codes deposited in GenBank were utilized to produce sequences in this study.**

| Accession Codes | Title |
| --- | --- |
| MW055178.1 | Lyssavirus rabies isolate GOA_A_25-04-2017 nucleoprotein (N) gene, complete cds |
| MW055159.1 | Lyssavirus rabies isolate GOA_B_15-12-2017 nucleoprotein (N) gene, complete cds |
| MW055148.1 | Lyssavirus rabies isolate GOA_B_15-11-2017 nucleoprotein (N) gene, complete cds |
| MW055174.1 | Lyssavirus rabies isolate GOA_A_02-01-2017 nucleoprotein (N) gene, complete cds |
| MW055173.1 | Lyssavirus rabies isolate GOA_A_22-05-2017 nucleoprotein (N) gene, complete cds |
| MW055189.1 | Lyssavirus rabies isolate GOA_A_14-11-2016 nucleoprotein (N) gene, complete cds |
| MW055164.1 | Lyssavirus rabies isolate GOA_B_04-03-2018 nucleoprotein (N) gene, complete cds |
| MW055171.1 | Lyssavirus rabies isolate GOA_A_30-12-2016 nucleoprotein (N) gene, complete cds |
| MW055160.1 | Lyssavirus rabies isolate GOA_A_31-08-2017 nucleoprotein (N) gene, complete cds |
| MW055163.1 | Lyssavirus rabies isolate GOA_A_06-04-2017 nucleoprotein (N) gene, complete cds |
| MW055184.1 | Lyssavirus rabies isolate GOA_A_13-02-2017 nucleoprotein (N) gene, complete cds |
| MW055179.1 | Lyssavirus rabies isolate GOA_A_26-12-2016 nucleoprotein (N) gene, complete cds |
| MW055183.1 | Lyssavirus rabies isolate GOA_A_11-01-2017 nucleoprotein (N) gene, complete cds |
| MH258816.1 | Lyssavirus rabies isolate India/dog/MVC-29/2014 nucleoprotein (N) mRNA, complete cds |
| MW055202.1 | Lyssavirus rabies isolate GOA_A_26-09-2016 nucleoprotein (N) gene, complete cds |
| MW055200.1 | Lyssavirus rabies isolate GOA_B_10-01-2017 nucleoprotein (N) gene, complete cds |
| MW055201.1 | Lyssavirus rabies isolate GOA_A_22-12-2017 nucleoprotein (N) gene, complete cds |
| MW055203.1 | Lyssavirus rabies isolate GOA_A_04-01-2018 nucleoprotein (N) gene, complete cds |
| MW055204.1 | Lyssavirus rabies isolate GOA_A_29-03-2017 nucleoprotein (N) gene, complete cds |
| MW055194.1 | Lyssavirus rabies isolate GOA_A_24-09-2017 nucleoprotein (N) gene, complete cds |
| MW055193.1 | Lyssavirus rabies isolate GOA_A_30-01-2018 nucleoprotein (N) gene, complete cds |
| MW055199.1 | Lyssavirus rabies isolate GOA_A_03-02-2018 nucleoprotein (N) gene, complete cds |
| MW055213.1 | Lyssavirus rabies isolate GOA_A_04-03-2018 nucleoprotein (N) gene, complete cds |
| KX434505.1 | Lyssavirus rabies isolate IMA-R146 nucleoprotein (N) gene, complete cds |
| KX434489.1 | Lyssavirus rabies isolate IAP-R91 nucleoprotein (N) gene, complete cds |
| KX434522.1 | Lyssavirus rabies isolate IGR-R206 nucleoprotein (N) gene, complete cds |
| KX434512.1 | Lyssavirus rabies isolate IAP-R192 nucleoprotein (N) gene, complete cds |
| KX434497.1 | Lyssavirus rabies isolate IKE-R111 nucleoprotein (N) gene, complete cds |
| KX434502.1 | Lyssavirus rabies isolate IKA-R132 nucleoprotein (N) gene, complete cds |
| KX434504.1 | Lyssavirus rabies isolate IKA-R144 nucleoprotein (N) gene, complete cds |
| KX434501.1 | Lyssavirus rabies isolate IKA-R129 nucleoprotein (N) gene, complete cds |
| KX434485.1 | Lyssavirus rabies isolate IKE-R78 nucleoprotein (N) gene, complete cds |
| KX434500.1 | Lyssavirus rabies isolate IKE-R121 nucleoprotein (N) gene, complete cds |
| KX434483.1 | Lyssavirus rabies isolate IKE-R73 nucleoprotein (N) gene, complete cds |
| KX434490.1 | Lyssavirus rabies isolate IKE-R94 nucleoprotein (N) gene, complete cds |
| KX434487.1 | Lyssavirus rabies isolate IKE-R87 nucleoprotein (N) gene, complete cds |
| KX434484.1 | Lyssavirus rabies isolate IKE-R77 nucleoprotein (N) gene, complete cds |
| KX434495.1 | Lyssavirus rabies isolate IKE-R109 nucleoprotein (N) gene, complete cds |
| KX434486.1 | Lyssavirus rabies isolate IKE-R86 nucleoprotein (N) gene, complete cds |
| KX434520.1 | Lyssavirus rabies isolate IGR-R204 nucleoprotein (N) gene, complete cds |
| KM979366.1 | Rabies virus strain NPBUF12-1 nucleoprotein gene, complete cds |
| JX944574.1 | Rabies virus isolate 3878-78(11008NEP) nucleoprotein (N) gene, complete cds |
| KX434509.1 | Lyssavirus rabies isolate IMA-R189 nucleoprotein (N) gene, complete cds |
| KX434510.1 | Lyssavirus rabies isolate IAP-R190 nucleoprotein (N) gene, complete cds |
| KX434511.1 | Lyssavirus rabies isolate IAP-R191 nucleoprotein (N) gene, complete cds |
| KX434516.1 | Lyssavirus rabies isolate IAP-R196 nucleoprotein (N) gene, complete cds |
| MW055133.1 | Lyssavirus rabies isolate A17-npval35 nucleoprotein (N) gene, complete cds |
| MW055134.1 | Lyssavirus rabies isolate GOA_A_10-03-2017 nucleoprotein (N) gene, complete cds |
| KX434519.1 | Lyssavirus rabies isolate IGR-R203 nucleoprotein (N) gene, complete cds |
| KM099393.1 | Rabies virus isolate RV-IGU-R202-Mongoose nucleoprotein (N) gene, complete cds |
| KF660246.1 | Rabies virus isolate IGU-200 nucleoprotein (N) gene, complete cds |
| KX434523.1 | Lyssavirus rabies isolate IGR-R207 nucleoprotein (N) gene, complete cds |
| AB910532.1 | Rabies virus N gene for nucleoprotein, complete cds, strain: Btn115 |
| JX944567.1 | Rabies virus isolate 3878-09(11009NEP) nucleoprotein (N) gene, complete cds |
| AB699215.1 | Rabies virus N gene for nucleocapsid, complete cds, strain: BDR2 |
| AB699216.1 | Rabies virus N gene for nucleocapsid, complete cds, strain: BDR3 |
| AB699214.1 | Rabies virus N gene for nucleocapsid, complete cds, strain: BDR1 |
| AB699219.1 | Rabies virus N gene for nucleocapsid, complete cds, strain: BDR7 |
| AB699218.1 | Rabies virus N gene for nucleocapsid, complete cds, strain: BDR6 |
| KX434517.1 | Lyssavirus rabies isolate IUP-R197 nucleoprotein (N) gene, complete cds |
| HE801588.1 | Rabies virus N gene for nucleoprotein, viral cRNA, isolate Pk 57 |
| JX987736.1 | Rabies virus isolate 04029AFG nucleoprotein (N) gene, complete cds |
| HE801584.1 | Rabies virus N gene for nucleoprotein, viral cRNA, isolate Pk 26 |
| HE801586.1 | Rabies virus N gene for nucleoprotein, viral cRNA, isolate Pk 55 |
| HE801580.1 | Rabies virus N gene for nucleoprotein, viral cRNA, isolate Pk 20 |
| HE801579.1 | Rabies virus N gene for nucleoprotein, viral cRNA, isolate Pk 19 |

**References:**

1. WSPA. World Society for the Protection of Animals (2009) Surveying roaming dog populations: guidelines on methodology. Companion & Working Animals Unit. World Society for the Protection of Animals, 89 Albert Embankment. London., 2009.

2. Iddrisu A-K, Besing Karadaar I, Gurah Junior J, Ansu B, Ernest D-A. Mixed effects logistic regression analysis of blood pressure among Ghanaians and associated risk factors. *Scientific Reports* 2023; **13**(1): 7728.

3. Reyment RA, Jvreskog K. Applied factor analysis in the natural sciences: Cambridge University Press; 1996.

4. Tryon RC. Cumulative communality cluster analysis. *Educational and Psychological Measurement* 1958; **18**(1): 3-35.

5. Chitra A, Rajkumar A. Paraphrase extraction using fuzzy hierarchical clustering. *Applied Soft Computing* 2015; **34**: 426-37.
